# Production and Purification of Filovirus Glycoproteins in Insect and Mammalian Cell Lines

**DOI:** 10.1038/s41598-017-15416-3

**Published:** 2017-11-08

**Authors:** Elizabeth C. Clarke, Amanda L. Collar, Chunyan Ye, Yíngyún Caì, Eduardo Anaya, Derek Rinaldi, Britney Martinez, Sarah Yarborough, Christine Merle, Manfred Theisen, Jiro Wada, Jens H. Kuhn, Steven B. Bradfute

**Affiliations:** 1 Center for Global Health, Division of Infectious Diseases, Department of Internal Medicine, University of New Mexico, Albuquerque, New Mexico, 87131 USA; 2 Integrated Research Facility at Fort Detrick, National Institute of Allergy and Infectious Diseases, National Institutes of Health, Frederick, Maryland 21702 USA; 3 Department of Pathology, University of New Mexico, Albuquerque, New Mexico, 87131 USA; 4 Undergraduate Pipeline Network, University of New Mexico, Albuquerque, New Mexico, 87131 USA; 5 Proteodynamics SARL, Riom, France

## Abstract

Filoviruses are highly virulent pathogens capable of causing severe disease. The glycoproteins of filoviruses are the only virally expressed proteins on the virion surface and are required for receptor binding. As such, they are the main candidate vaccine antigen. Despite their virulence, most filoviruses are not comprehensively characterized, and relatively few commercially produced reagents are available for their study. Here, we describe two methods for production and purification of filovirus glycoproteins in insect and mammalian cell lines. Considerations of expression vector choice, modifications to sequence, troubleshooting of purification method, and glycosylation differences are all important for successful expression of filovirus glycoproteins in cell lines. Given the scarcity of commercially available filovirus glycoproteins, we hope our experiences with possible difficulties in purification of the proteins will facilitate other researchers to produce and purify filovirus glycoproteins rapidly.

## Introduction

Filoviruses (mononegaviral family *Filoviridae*) are a group of highly virulent pathogens that can cause viral hemorrhagic fevers in humans and/or nonhuman primates. Currently, eight recognized filoviruses are classified into three genera: *Cuevavirus*, *Ebolavirus*, and *Marburgvirus* ^1^. The members of the genus *Ebolavirus*, i.e., ebolaviruses, are Bundibugyo (BDBV), Ebola virus (EBOV), Reston virus (RESTV), Sudan virus (SUDV), and Taï Forest virus (TAFV). All ebolaviruses except RESTV cause Ebola virus disease (EVD) in humans. The genus *Marburgvirus*, i.e., marburgviruses, contains Marburg virus (MARV) and Ravn virus (RAVV), both of which cause Marburg virus disease (MVD) in humans^2^. Finally, the genus *Cuevavirus* has a single member, Lloviu virus (LLOV), which has been associated with lethal disease in bats but has unknown pathogenicity for primates^3^.

**Figure 1:**
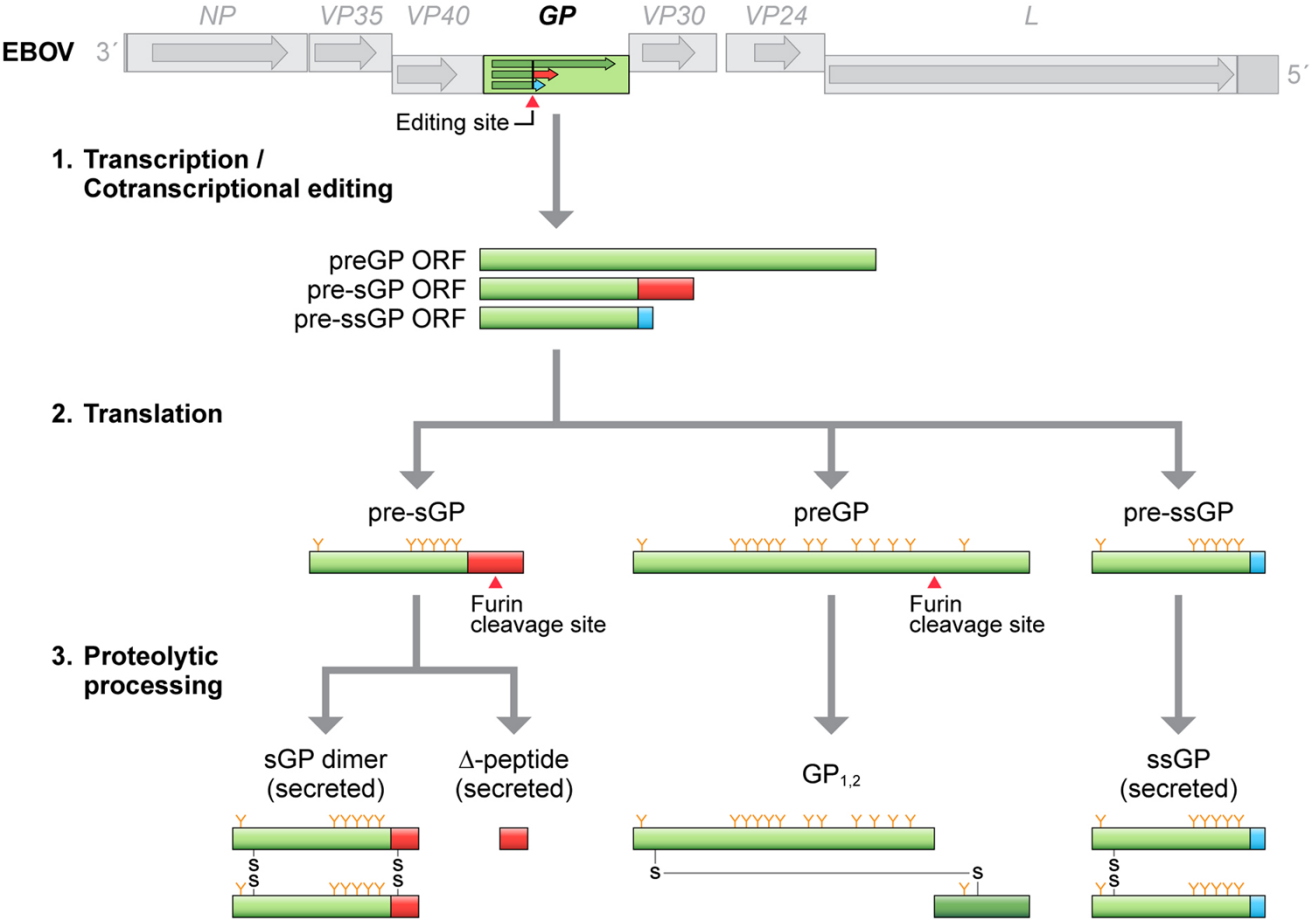
Schematic of the Ebola virus (EBOV) *GP* gene expression strategy. Primary (unedited) transcription of the *GP* gene results in an mRNA leading to the expression of pre-sGP. pre-sGP is proteolytically cleaved by furin into mature and homodimerized secreted glycoprotein (sGP) and secreted Δ-peptide. EBOV RNA-dependent RNA polymerase (L) stuttering at a 7U-editing site within the *GP* gene infrequently results in the addition or subtraction of cognate A residues into nascent mRNAs, thereby disrupting the sGP open reading frame (ORF) and joining the sGP ORF upstream of the editing site with overlapping ORFs downstream. mRNAs with an 8A editing site result in the expression of preGP. preGP is proteolytically cleaved by furin into subunits GP_1_ and GP_2_, which remain connected through a disulfide bond in the form of a heterodimer (GP_1,2_). mRNAs with a 6A or 9A editing site result in expression of pre-ssGP, which is proteolytically matured into homodimeric secondary secreted glycoprotein (ssGP). The GP expression strategies of other ebolaviruses and of cuevaviruses follow the same pattern as that of EBOV. Marburgvirus *GP* genes, on the other hand, only contain a single ORF encoding GP_1,2_. Orange-colored Y’s signify glycosylations.

Filovirions enter target cells through interaction of their only particle surface protein, glycoprotein GP_1,2_, with cell-surface attachment factors and Niemann Pick C1 (NPC1) as the common endosomal entry receptor^4,5^. GP_1,2_, is a typical class I fusion type 1 transmembrane protein that is highly glycosylated and serves as the primary target for neutralizing antibodies^6–8^. GP_1,2_ is expressed from the fourth of the seven filoviral genes, *GP*. In the case of marburgviruses, GP_1,2_ is the only *GP* expression product. In the case of cuevaviruses and ebolaviruses, the primary *GP* expression product is a non-structural secreted glycoprotein of unknown function (sGP). GP_1,2_ and another non-structural secreted glycoprotein (ssGP) are expressed via co-transcriptional mRNA editing resulting in addition of one or two adenosyls into the mRNA, respectively, thereby leading to open reading frame switches^9–12^ (Fig. 1). Filoviral GP_1,2_ is expressed akin to typical preproproteins. The primary expression product is steered into the endoplasmic reticulum (ER) by its signal peptide. Signalase cleaves off the signal peptide to yield preGP, and a host protease, furin, cleaves preGP into two subunits, GP_1_ and GP_2_, that remain linked by disulfide bonds (GP_1,2_)^8,13,14^.

Despite the virulence of filoviruses, most filoviruses are not thoroughly characterized, and comparatively few commercially produced reagents are available for their study^15,16^. For instance, availability of soluble filovirus GP_1,2_ is necessary for a variety of applications, including greater understanding of GP_1,2_-receptor or GP_1,2_-antibody binding kinetics, vaccine development, and GP_1,2_ structural studies. An ectodomain of the EBOV (variant Yambuku, isolate Mayinga) GP_1,2_ has been used as part of commercially available ELISAs for quantitation of antibody responses^17^, and a soluble, modified EBOV Yambuku-Mayinga GP_1,2_ ectodomain has been used to determine the crystal structure of GP_1,2_ bound to NPC1^18^. The ectodomain of both EBOV^8^ and MARV^19^ with mucin-like domain deletions have been produced previously for crystallization studies. Additionally, efforts such as vaccine development use different organismal cell types as platforms to produce filovirus GP_1,2_, including mammalian cells^20–22^ and insect cells^23^. Specifically, authors have successfully used the Sf9-baculovirus system to produce full-length Ebola glycoproteins for use in VLPs^23^ and nanoparticle vaccines^24,25^. Other groups have also successfully used poly-histidine (6xHis) tags to purify full-length EBOV glycoproteins^26^. Some insect-derived filovirus (predominantly EBOV) GP_1,2_s are commercially available, but most mammalian-derived filovirus GP_1,2_s are not.

To close gaps in filovirus GP_1,2_ availability, we report on two systems for production and purification of filovirus GP_1,2_s in insect (Sf9) and mammalian (human) cell lines, respectively. Using these systems, we have successfully expressed EBOV, BDBV, TAFV, SUDV, MARV, and LLOV GP_1,2_s and developed techniques for rapid production of soluble variants thereof. We recently used these techniques to successfully produce ebolavirus GP_1,2_s for glycosylation analysis of their glycans^27^. Our expression systems may be broadly applicable for production and affinity purification of other soluble proteins from insect and mammalian cells.

We also demonstrate here that the ebolavirus GP_1,2_ proteins obtained using the two systems have important differences in glycosylation. These differences encompass the number of glycans, the type of glycan species, and the distribution of glycans at specific sites. We consider that these data to have implications for downstream usages of the produced proteins, such as binding assays, where glycosylation of the proteins may impact function.

## Results

### Modification of filovirus GP_1,2_ sequences

**Figure 2:**
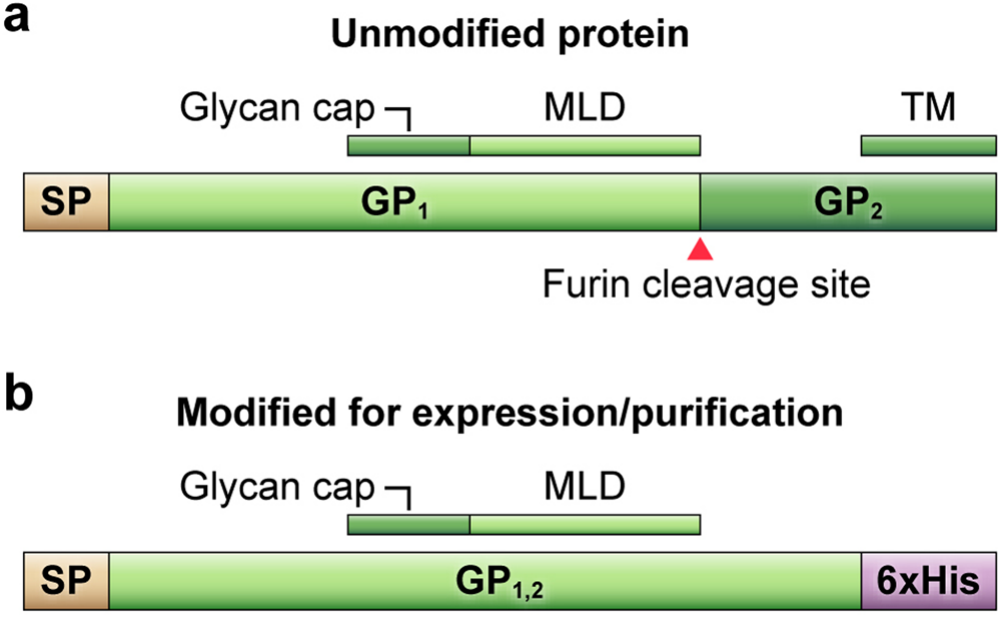
Modifications made to the filovirus *GP* genes for expression and purification of GP_1,2_s. (**a**) *GP* gene co-transcriptional mRNA editing results in an 8A mRNA encoding a preproprotein consisting of a signal peptide (SP) and preGP (later cleaved by furin into subunits GP_1_ and GP_2_). The GP_1_ portion of preGP contains a glycan cap and mucin-like domain (MLD), whereas the GP_2_ portion of the protein anchors the GP_1,2_ trimer in membranes via a transmembrane domain (TM). (**b**) Constructs used for this study are expressed from engineered genes to only express the GP_1,2_ ORF in the absence of the furin cleavage site and TM replaced with 6xHis tag.

We made changes to the sequence of the filovirus glycoproteins of interest to aid in expression and purification, including: mutating the furin cleavage site for purification of GP_1,2_ complexes; truncating the GP_2_s upstream of the transmembrane domain to produce soluble GP_1,2_ complexes; mutating the editing site of the *GP* genes to ensure exclusive expression of GP_1,2_ complexes; and adding 6xHis tags to the C-termini of GP_2_s for purification GP_1,2_ complexes (Fig. 2).

### Generation of baculoviruses containing modified filovirus GP_1,2_s

*GP* genes were synthesized with the modifications discussed above and cloned into pFastBac1 plasmids to generate bacmids for transfection. After transfection of bacmids into the Sf9 cells, expressed GP_1,2_s produced were verified by western blot and plaque purified to ensure that clonal baculovirus was used for infections.

### Purification of Sf9-produced GP_1,2_s using nickel columns

As the GP_1,2_s produced by the baculovirus-infected Sf9 cells do not contain a transmembrane region, all GP_1,2_s were released into Sf9 culture media. Total media were collected at day 3 post-transfection, replaced, collected again at day 4, and pooled together for purification. For each purification, between 2–4 T150 flasks of Sf9 cells were used for overall final yields ranging from 400 µg to 800 µg of GP_1,2_.

**Figure 3:**
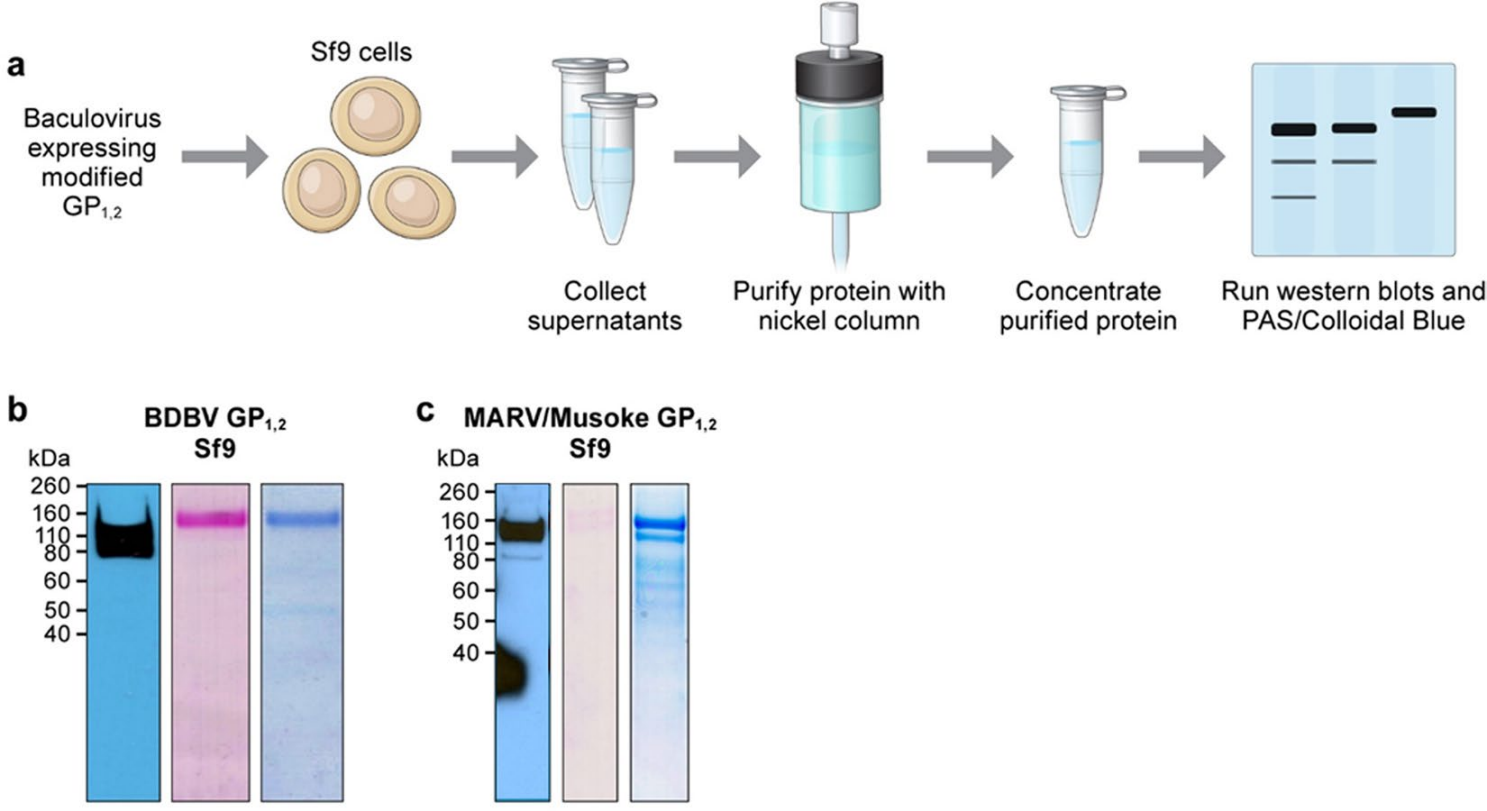
Western blot gels verifying the purity of modified GP_1,2_s obtained from Sf9 insect cells. (**a**) Schematic of the Sf9 filovirus GP_1,2_ production workflow. (**b**) Western blot, PAS, and colloidal blue staining of purified BDBV GP_1,2_ from Sf9 cells. (**c**) Western blot, PAS, and colloidal blue staining of MARV GP_1,2_.

Purified proteins were verified by western blot using antibodies against either the specific GP_1,2_, or against the His-tag in the case of LLOV GP_1,2_. Purity was confirmed by periodic acid-Schiff (PAS) staining (preservation of glycosylation patterns) and by Colloidal Blue staining for detection of overall protein. Using this method of production, high levels of GP_1,2_ purity were obtained with the nickel column (Fig. 3b,c, Supplementary Fig. S1).

Additionally, we attempted protein production in a different insect cell line, High Five cells (ATCC CRL-10859), which have been reported^28^ to produce greater amounts of protein upon infection with baculoviruses. In our small-scale experiments, High Five cells did not yield greater protein than Sf9 cells, but this yield is likely to protein specific (data not shown).

### Expression of filovirus GP_1,2_ in HEK 293T cells

Modified *GP* genes were cloned into pcDNA3.1^+^ backbones using the CMV enhancer-promoter to drive high levels of mammalian expression. BDBV, EBOV/Makona, EBOV/Yambuku, SUDV, TAFV, and LLOV GP_1,2_s were all successfully produced 3 days after Jet Prime transfection of the pcDNA3.1^+^ vector.

### Plasmid vector backbone affects expression level of proteins

In our experiments, MARV/Musoke GP_1,2_ could not be expressed using pcDNA3.1^+^. The sequence of the MARV/Musoke *GP* gene used was truncated at residues encoding amino acid 636 (preGP numbering), i.e., upstream of the predicted transmembrane region^29^.This sequence was identical to the sequence used in the MARV/Musoke *GP* gene cloned into the baculovirus that successfully produced MARV GP_1,2_ in the insect cell system. Likewise, expression of the MARV/Musoke *GP* gene encoding a GP_1,2_ truncated to encode only amino acid residues 1–648 or 1–644 was unsuccessful in the pcDNA3.1^+^ background (Table 1). In contrast to commercially available MARV GP_1,2_ expression plasmids, which use codon-optimized *GP* genes for expression in mammalian cells, all our modified sequences were based on the wild-type *GP* sequences. Transfer of the MARV/Musoke *GP* gene sequence from pcDNA3.1^+^ into the pCAGGs expression vector did not lead to successful expression of MARV GP_1,2_, despite successful expression of unmodified full-length MARV *GP* by pCAGGs and not pcDNA3.1^+^ (Table 1, Supplementary Fig. S2). However, MARV/Angola (1–648) GP_1,2_ could be successfully expressed from pCAGGs (Table 1, Supplementary Fig. S2).

**Table 1:** Expression attempts of various genes encoding MARV GP_1,2_ constructs from different expression vectors.

Expression vector	Expressed constructs	HEK 293T expression
pcDNA3.1^+^	full-length MARV/Musoke GP_1,2_	No
pcDNA3.1^+^	MARV/Musoke GP_1,2_ (1–648)^a^	No
pcDNA3.1^+^	MARV/Musoke GP_1,2_ (1–644)^a^	No
pcDNA3.1^+^	MARV/Musoke GP_1,2_ (1–636)^a^	No
pCAGGS	full-length MARV/Musoke GP_1,2_	Yes
pCAGGS	MARV/Musoke GP_1,2_ (1–636)^a^	No
pCAGGS	MARV/Angola GP_1,2_ (1–648)^a^	Yes

^a^Parentheses contain amino-acid lengths of different constructs. Expression data shown in Supplementary Fig. 2.

### Mammalian HEK 293T culture conditions are incompatible with nickel column purification

**Figure 4:**
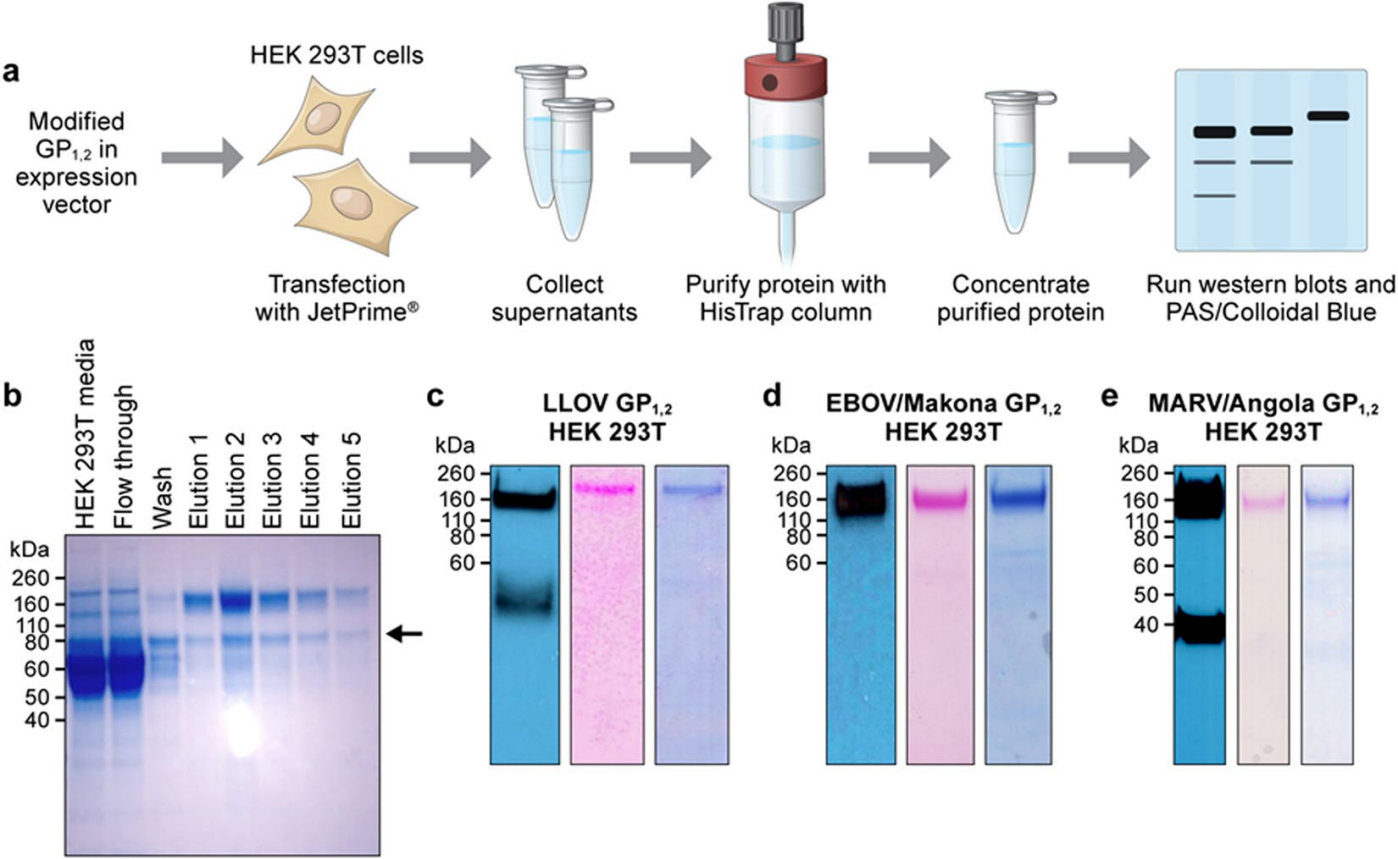
Western blots verifying purity of GP_1,2_s obtained from HEK 293T cells. (**a**) Schematic of the filovirus GP_1,2_ production workflow from HEK 293T cells. (**b**) EBOV/Makona GP_1,2_ from HEK 293T cells was loaded onto a HisTrap Excel column. A contaminant was detected by western blot and PAS and colloidal blue stains, as shown by an arrow, of the elutes from the column of media only, flow through wash, and five 1-minute dilutions. (**c**) Western blot and PAS and colloidal blue staining of LLOV GP_1,2_ from HEK 293T cells. (**d**) Western blot and PAS and colloidal blue staining of purified EBOV/Makona GP_1,2_ from HEK 293T cells. (**e**) Western blot and PAS and colloidal blue staining of MARV-Angola GP_1,2_ from HEK 293T cells.

After verifying successful filovirus GP_1,2_ expression from plasmids, we transfected plasmids into HEK 293T cells for purification and harvested media on day 3 post-transfection. Initially, we again used the HisTrap Excel Nickel Column for purification. However, elutions from the column contained an additional contaminating band (Fig. 4b) of approximately 80 kDa. This protein was glycosylated, as determined by PAS staining, but did not react with antibodies against GP_1,2_.

We attempted to remove the contaminant through multiple methods. Initial attempts to modify the HisTrap Excel protocol by doubling the wash buffer volume, increasing imidazole concentration in wash buffer, and increasing imidazole concentration in sample did not eliminate the contaminant. We used 8 M of urea to dissociate a possible protein-protein interaction with the contaminant. However, running the supernatant on a native gel revealed that the contaminating protein was not interacting with the GP_1,2_s. Due to the size difference between the GP_1,2_s and the contaminant, we also attempted dialysis of the elution with membranes with pore sizes of 100 kDa, 300 kDa, or 1,000 kDa. We then used size exclusion columns to remove the contaminant, but without success. We tried altering the purification method first by using a His-streptavidin tag-enrichment kit with a streptavidin column and then by replacing the nickel column with a cobalt column. Both approaches have been reported to reduce binding of contaminating proteins due to lower affinity for the His-tag. Yet, these approaches were not effective. The contaminating protein was identified by mass spectrometry as bovine serotransferase, a component of the fetal bovine serum (FBS) used in the HEK 293T cell media.

### Purification of HEK 293T cell-produced GP_1,2_s with anti-His affinity resin columns

Anti-His Affinity Resin (GenScript) was used for purification of filovirus GP_1,2_ to avoid bovine serotransferase protein contamination. T150 flasks containing HEK 293T cells at 50–80% confluency were transfected with plasmids and, on day 3 post-transfection, media were collected for use in purification. Each purification on the Anti-His Affinity Resin gave an overall protein yield ranging from 150 µg to 500 µg, depending on GP_1,2_. Different *GP* genes resulted in different GP_1,2_ expression levels, with EBOV/Makona and EBOV/Yambuku genes expressing more GP_1,2_s than those of other filoviruses in both insect and mammalian cells (data not shown). Purified GP_1,2_s were again verified by western blot, and purity was confirmed by PAS and Colloidal Blue staining (Fig. 4c–e). Using this approach for glycan analysis, we confirmed the uniformity of the individual GP_1,2_s produced, demonstrating consistent GP_1,2_ glycosylation over multiple batches^27^.

### Enzymatic digest of glycans from HEK 293T and SF9-cell produced GP_1,2_

**Figure 5:**
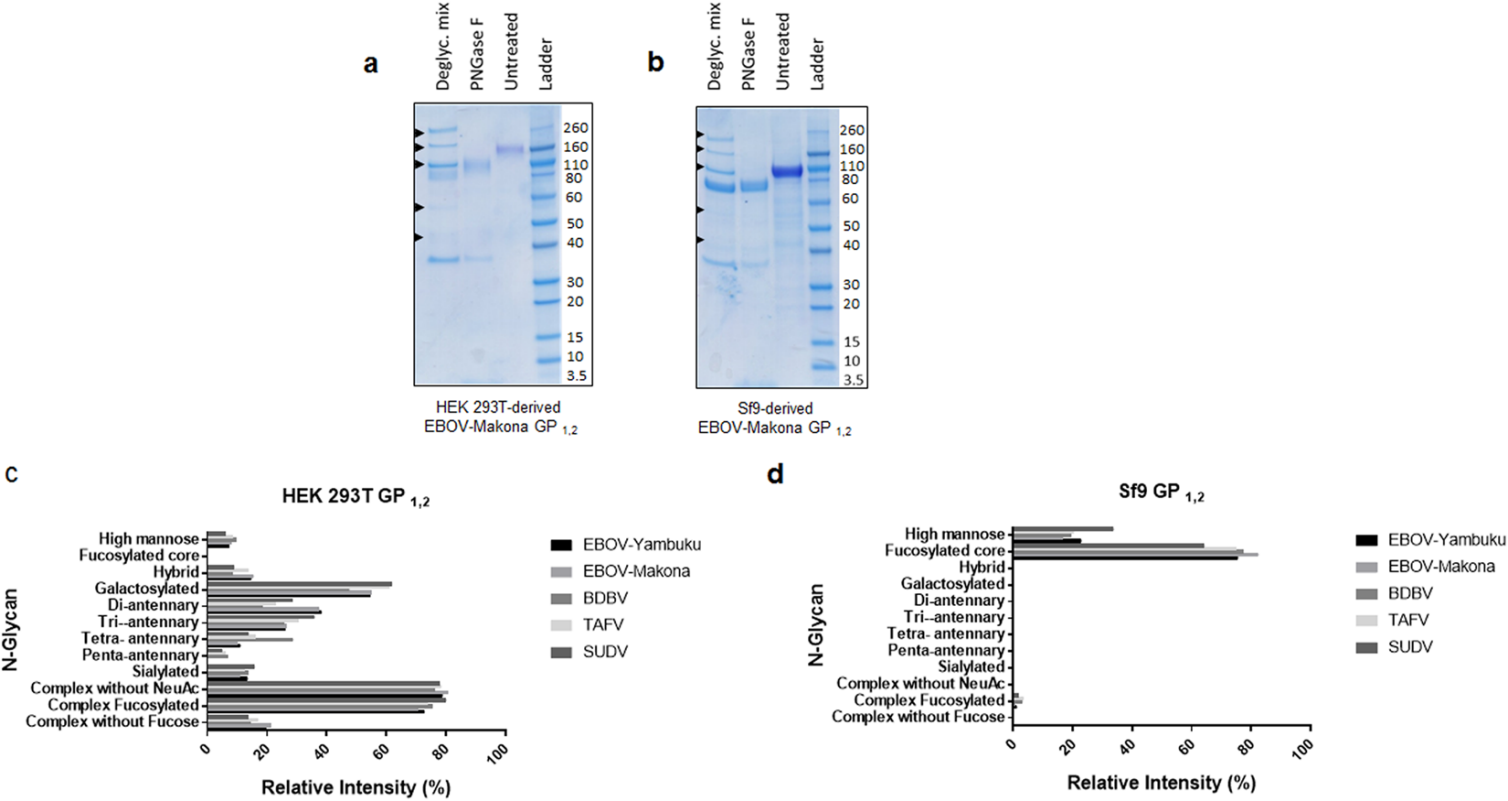
Enzymatic removal of glycans and N-linked glycan type analysis. (**a**,**b**) EBOV/Makona GP_1,2_ from HEK 293T cells (**a**) or Sf9 cells (**b**) digested with PNGase F to remove N-linked glycans and deglycosylation mix to remove the majority of O and N-linked glycans, shown by colloidal blue stain. Arrows indicate non- GP_1,2_proteins from the deglycosylation mix itself and protein ladder indicates sizes of intact and digested GP_1,2_ bands. (**c,d**) N-linked glycans revealed by MALDI-TOF MS from ebolavirus GP_1,2_s produced in HEK293T cells (**c**) and Sf9 cells (**d**) grouped by glycan type. Portions of the data in (**c**) showing levels of High Mannose, Hybrid, Galactosylated, Sialylated, Complex without NeuAc, Complex With NeuAc, With Fucosylation, and Without Fucosylation were published previously^27^.

EBOV/Makona GP_1,2_ produced in HEK 293T cells has a molecular weight by electrophoresis of around 160kDa, compared to just ≈110 kDa molecular weight of Sf9-produced EBOV/Makona (Fig. 5a,b). To demonstrate the contribution to molecular weight from glycosylation, we removed N-linked glycans from both proteins using PNGase F, and showed that ≈31% of 160 kDa HEK 293T cell-derived EBOV/Makona GP_1,2_, or 50 kDa total molecular weight, are N-linked glycans, compared to ≈35%, or ≈38 kDa of 110 kDa Sf9 cell-derived EBOV/Makona GP_1,2_. We then used a deglycosylation mix of enzymes that removes the majority of N and O-linked glycans and found that an additional ≈24%, or 38  kDa, of the HEK 293T cell-derived protein are O-linked glycans. However, the deglycosylation enzyme mix did not further reduce the molecular weight of the Sf9-derived EBOV/Makona protein, suggesting little or no observable O-linked glycosylation on the insect cell derived-proteins.

### Analysis of N-linked glycans in HEK 293T and Sf9 cell produced GP_1,2_s

Our group has previously published in detail on the composition of the N-glycans on ebolavirus GP_1,2_^27^. The broad differences in ebolavirus GP_1,2_ produced in HEK 293T cells are summarized in Fig. 5c. Briefly, the majority of the N-linked glycans are of the complex type, with few high mannose and hybrid glycans. The majority of the glycan species imparted on the mammalian GP_1,2_s are fucosylated. Di-antennary N glycans which are the dominant complex glycan structures for both EBOV GP_1,2_ samples are significantly less represented for BDBV, TAFV and SUDV GP_1,2_. The latter proteins showed increased levels of tri- tetra-, and penta- antennary N-glycans. Sialylated N-glycans correspond in all HEK 293T derived GP_1,2_ samples to about 15% of total N-glycans. Analysis of the Sf9-cell derived ebolavirus GP_1,2_ proteins reveals simpler glycan profiles, with a fucosylated core N-glycan (Fuc)1 (Man)3 –GlcNAc)2 as the largely dominant structure and some high-mannose type glycans (Fig. 5d). The N-glycans found on Sf9 cell derived GP 1,2 were in accordance with the glycosylation potential of this expression system^30^ lacking notably complex galactosylated or sialylated glycans.

### Analysis of O-linked glycans in HEK293T and Sf9 cell-produced GP_1,2_s

The GP_1,2_s of filoviruses are predicted to have extensive O-linked glycosylation in addition to N-linked glycosylation. We have previously shown that ebolavirus GP_1,2_s produced in HEK 293T cells are extensively glycosylated with O-linked glycans, although the individual O-glycans imparted vary among different ebolaviruses, including between the two strains of EBOV, Makona and Yambuku^27^. To investigate the differences between GP_1,2_s produced in insect and mammalian cell types, we analyzed O-linked glycosylation of Sf9-derived ebolavirus GP_1,2_s. Primary analysis of ebolavirus GP_1,2_s generated in insect cells by mass spectrometry analysis of released permethylated O-glycans did not reveal any classical O-glycans for EBOV/Makona, EBOV/Yambuku, BDBV, TAFV and SUDV GP_1,2_s. However, monosaccharide quantification of EBOV Makona GP_1,2_ from Sf9 cells was performed to verify the presence of *N-*acetylgalactosamine (GalNAc) residues. These residues might be indicative of the presence of simple O-glycan structures on the protein that had not been detectable by O-glycan profiling of permethylated glycans. The observed presence of a low amount of GalNAc (measured as Galactosamine, GalN) of 3.3 nmol per nmol of protein allows us to presume that EBOV Makona from Sf9 cells carries a simple O-glycosylation essentially consisting of GalNAc residues. The presence of 1nmol of galactose per nmol of protein may indicate that some (Gal)_1_ (GalNAc)_1_ structures are present. These data suggest that insect-derived filovirus GP_1,2_ carry minimal levels of O-glycans that consist primarily of single GalNAc residues, in comparison to the extensively O-glycosylated HEK 293T-derived ebolavirus GP_1,2_s.

### N-glycan site occupancy for EBOV/Makona GP_1,2_ produced in HEK 293T cells and Sf9 cells

**Figure 6:**
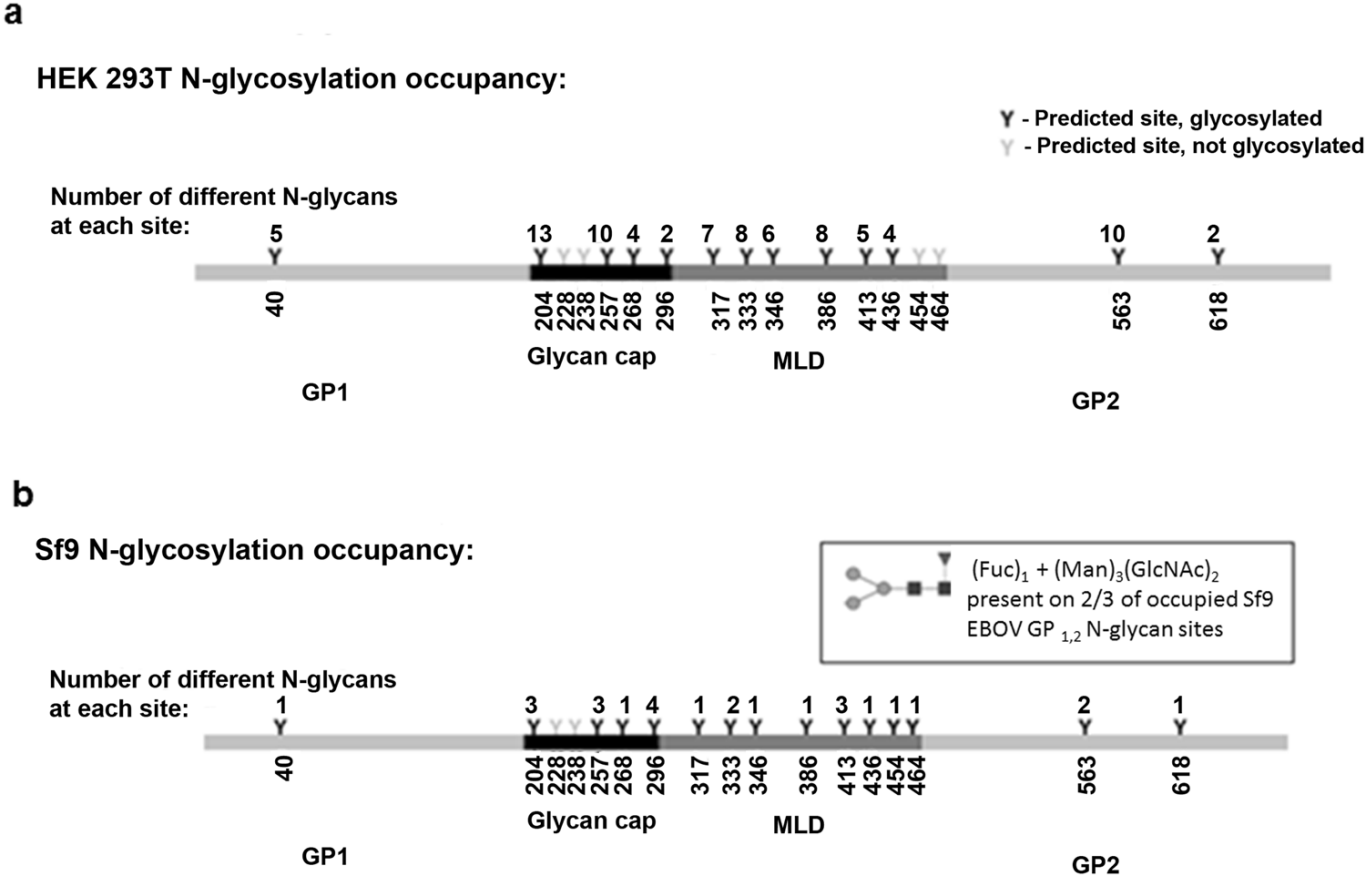
Schematic of site occupancy analysis of N-linked glycans on EBOV/Makona GP_1,2_ produced in HEK 293T cells (**a**) and Sf9 cells (**b**). Predicted sites determined using open-source EXPAZY Glycomod and the EBOV/Makona amino acid sequence.

The GP_1,2_ protein of EBOV/Makona contains 17 potential N-glycosylation sites that are represented in Fig. 6, obtained by analysis of the sequence using database search on EXPAZY Glycomod. The prediction of glycosylation sites does not differentiate based on cell or organism type but is only based on the presence of the N-glycosylation sequon Asn -X-Ser/Thr within the protein sequence. In a preliminary study combining the analysis of glycopeptides prior to and after enzymatic glycosylation, occupation of most of the 17 theoretical N-glycosylation sites with N-glycans could be observed. Sf9-derived EBOV/Makona GP_1,2_ contained15/17 detectable occupied sites while HEK 293T-derived EBOV/Makona GP_1,2_, revealed the presence of 13/17 detectable occupied sites. At the present level of analysis we cannot exclude that the remaining theoretical glycosylation sites are also occupied since the relatively large sizes of the corresponding tryptic glycopeptides may have prohibited their analysis by MALDI-TOF mass spectrometry. There are significant differences in the variety of different glycan species found at any given N-glycan occupied site, with the HEK 293T-derived EBOV/Makona GP_1,2_ having a greater number, up to 13 different N-glycans at an individual site (N172 corresponding to amino acid 204 of the construct), compared to the Sf9-derived, which had a maximum of 4 N-glycans at a given site (N264 corresponding to amino acid 296 of the construct). The heterogeneity of site specific N-glycosylation between mammalian and insect cell derived GP_1,2_ is in accordance with the observed differences of overall N-glycan profiles of proteins produced in these expression systems. Significantly, the two predicted glycosylation sites in GP_1,2_ shown to be necessary for VSV pseudotype entry and production^31^ of full-length EBOV/Mayinga GP_1,2_, were also found to be glycosylated in our EBOV/Makona GP_1,2_ produced by either cell type.

## Discussion

In the present study, we demonstrate two techniques for producing and purifying filovirus GP_1,2_s. We modified the *GP* gene sequences from ebolaviruses, MARV, and LLOV to express released GP_1,2_s by truncating the sequences to remove the transmembrane domains. For higher expression, we altered the RNA-editing sites of the ebolavirus and LLOV *GP* genes (Fig. 2). This approach prevented expression of sGP and ssGP, which could interfere with growth of cells in culture and downregulate GP_1,2_ expression^32^. EBOV GP_1,2_ has been reported to be cytotoxic when expressed at high levels^33,34^, which would not be ideal for protein production. However, this property has been attributed to the transmembrane region of the protein^35^, and thus deleting the transmembrane domain may have circumvented this potential problem.

We mutated the furin cleavage sites of filovirus GP_1,2_s to prevent dissociation of the GP_1_ and GP_2_ subunits during the purification process (Fig. 2). This step enabled maintenance of equal expression levels of GP_1_ and GP_2_ and circumvented purification of only GP_2_ (which contained the His-tag). However, we did not codon-optimize the *GP* gene sequences because codon optimization is known to increase protein expression levels, thereby possibly leading to cytotoxicity^33,34^. The modified sequences were cloned into either pcDNA3.1^+^, a commonly used expression plasmid for mammalian cell transfections, or into a shuttle vector, pFastBac1, for baculovirus (AcMNPV) production. Insect cells are a commonly used system for protein production in part because of the success of baculovirus systems. Baculoviruses are versatile vectors for protein production in insect cells that are easily scaled up for high output^28,36^.

We successfully expressed modified ebolavirus and LLOV GP_1,2_s in HEK 293T cells using the pcDNA3.1^+^ expression plasmid. However, we were unable to successfully produce MARV GP_1,2_ in the HEK 293T cell system using pcDNA3.1^+^ (Table 1). Non-modified full-length MARV GP_1,2_ was also not successfully produced in the HEK 293T system using the pcDNA3.1^+^ vector but was produced using the vector pCAGGs despite the encoded sequences being identical. Further, using the pCAGGs expression vector, we could not express modified MARV/Musoke GP_1,2_, but could express modified MARV/Angola GP_1,2_ (Table 1, Supplemental Fig. 2). The amino acid sequence difference between the two glycoproteins is 7.2%^37^. Thus, the vector choice is gene-specific in the mammalian system, even among genes from related viruses. Therefore, attempting expression with multiple vectors may be necessary for a protein of interest.

Attempts to purify mammalian-derived GP_1,2_ through the HisTrap Excel Nickel Column system that we used for Sf9 purification were unsuccessful due to a component of the FBS that binds to the nickel column (Fig. 4b). This component was identified as bovine serotransferase. To our knowledge, this paper is the first report of an incompatibility of the nickel column system and mammalian tissue culture that uses FBS. The Anti-His Affinity Resin approach that we detail here may therefore be applicable to purification of many mammalian cell-produced proteins. Another alternative may be the use of serum-free 293T cell culture media. In summary, we demonstrated that different purification techniques are required for mammalian and insect filovirus GP_1,2_ production.

We demonstrate that there are key differences in the type of glycosylation imparted on the ebolavirus GP_1,2_s produced using the two methods outlined, for insect and mammalian cell culture. Key differences in complexity of the N-linked glycans between the two systems were found for all of the ebolavirus GP_1,2_s. The mammalian derived-proteins demonstrated high percentages of complex N-linked glycans compared to the majority of simple N-linked glycans and high mannose structures typically found in SF9 insect cell derived protein. The HEK 293T derived GP_1,2_s showed some heterogeneity in the complexity of antennary structures with the two EBOV samples containing predominantly di-antennary complex glycans and BDBV, TAFV and SUDV harboring complex glycans with higher antennary structures. The mammalian system also produced N-glycans that were sialylated. Although it remains controversial whether some insect protein production systems are able to impart sialylation, with some well controlled studies suggesting that they can at a minimal level^38^. Using our Sf9 cell production system, we did not see sialylation on any insect-derived N-linked glycans. As sialylation has been shown to play an important role in the function of some glycoproteins, it is possible that this difference could impart significant functional differences^39^.

Insect cell ability to impart O-linked glycans has been controversial, with some studies demonstrating limited O-linked glycans present on proteins derived from Sf9 cells^40^. Our analysis reveals the possibility of limited GalNAc-only O-linked glycans on the EBOV/Makona analyzed by monosaccharide quantification. However, while there is some evidence of limited O-linked glycosylation from insect cell-produced GP_1,2_s, mammalian cell O-linked glycosylation is extensive, a key difference between the two systems of production. For lesser studied ebolaviruses such as Bundibugyo and Taї Forest viruses, few reagents are available, and the few commercially available proteins are often produced in insect cells. We demonstrate that the post-translational modifications are different in mammalian compared to insect systems for production of filovirus GP_1,2_s. A comparison between the two systems may be useful in deciding how to produce large-scale amounts of protein for purposes such as vaccination, enzyme-linked immunosorbent assays, or the study of receptor or antibody binding kinetics. Our approaches successfully produced filovirus GP_1,2_s in both systems, therefore allowing phenotypic comparisons between the expressed proteins.

## Materials and Methods

### Cells and plasmid vectors

Fall armyworm (*Spodoptera frugiperda*) Sf9 cells (Invitrogen, Germany) were grown at 27 °C in Sf-900II serum-free medium (Gibco, USA) with 100 U/ml of penicillin and 100 µg/ml of streptomycin in the suspension. Human embryonic kidney (HEK) 293T cells (ATCC CRL-3216, USA) were grown in Dulbecco’s modified Eagle’s medium (DMEM, Gibco) supplemented with 10% heat-inactivated fetal calf serum (FCS) and 1% penicillin-streptomycin.

We created GP_1,2_-expressing plasmids based on *GP* genes from one cuevavirus, four ebolaviruses, and two marburgviruses. All but two plasmids are based on reference *GP* sequences: Lloviu virus/M.schreibersii-wt/ESP/2003/Asturias-Bat86 (“LLOV”; RefSeq #NC_016144), Bundibugyo virus/H.sapiens-tc/UGA/2007/Butalya-811250 (“BDBV”; RefSeq #NC_014373), Ebola virus/H.sapiens-tc/COD/1976/Yambuku-Mayinga (“EBOV/Yambuku”; RefSeq #NC_002549), EBOV/H.sapiens-wt/GIN/2014/Makona-C07 (“EBOV/Makona”, GenBank #KJ660347), Sudan virus/H.sapiens-tc/UGA/2000/Gulu-808892 (“SUDV”; RefSeq #NC_006432), Taï Forest virus/H.sapiens-tc/CIV/1994/Pauléoula-CI (“TAFV”; RefSeq #NC_014372), Marburg virus/H.sapiens-tc/KEN/1980/Mt. Elgon-Musoke (“MARV”; RefSeq #NC_001608), and Marburg virus/H.sapiens-tc/AGO/2005/Angola-20051379 (“MARV/Angola”, GenBank #KU978782). All genes were synthesized by Genscript (Piscataway, NJ) or cloned at Biosafety level 2 by the authors from inactivated filovirus cultures. All experiments were performed in Biosafety level 2 conditions.

### Glycoprotein sequence modifications

A number of *GP* gene modifications was required to produce soluble filovirus GP_1,2_s. An additional adenosyl was added to the cuevavirus and ebolavirus 7A co-transcriptional editing sites (yielding 8A-GP) to ensure expression of GP_1,2_ in the absence of sGP and ssGP production^41^. The coding regions for the GP_2_ transmembrane regions were removed to ensure secretion. Truncation occurred at amino acid 692 (LLOV preGP numbering), 650 (ebolavirus preGP numbering), and 638 (MARV preGP numbering). A region encoding a C-terminal polyhistidine (6xHis) tag was added to all genes to aid purification of expressed proteins through affinity chromatography. Finally, furin cleavage of preGPs was prevented by site-directed mutagenesis leading to a single arginine-to-lysine change in the VYFRRKR furin cleavage site. This change is known not to affect the protein’s function^42^, which is why we continue to refer to GP_1,2_s in this manuscript.

### Protein expression

Modified *GP* genes were cloned into the pcDNA3.1^+^ mammalian expression vector and transfected into HEK 293T cells at 50–80% confluency using JetPRIME transfection reagent (Polyplus transfection, New York, NY). Modified *GP* genes were also cloned into pFastBac™1 (ThermoFisher Scientific, Waltham, MA), a transposing vector with the baculovirus Autographa californica multicapside nucleopolyhedrovirus (AcMNPV) polyhedrin (PH) promoter. The pFastBac™1 constructs were then transformed in MAX Efficiency® DH10Bac™-competent cells (ThermoFisher Scientific, Waltham, MA), generating recombinant bacmids for transfection into Sf9 cells using Cellfectin® II Reagent (ThermoFisher Scientific, Waltham, MA). AcMNPVs expressing GP_1,2_s were verified for correct orientation 72 h after transfection of Sf9 cells in 6-well plates by western blot of cell supernatants. Supernatants containing AcMNPVs was then used for plaque purification with 10-fold dilutions. Individual plaques were purified by adding an agarose plug to a T150 Sf9 flask and incubating for 4 days. Supernatants from these flasks were titered using plaque assays of 95% confluent Sf9 cells and a 4% agarose overlay.

### Purification of Sf9-produced glycoproteins

Sf9 cells were infected with AcMNPVs at a multiplicity of infection of 0.01. Media were harvested on days 3 and 4 post-inoculation and pooled. Modified GP_1,2_s were purified using the HisTrap excel system (GE Healthcare Bio-Sciences, Pittsburgh, PA), following the manufacturer’s instructions. Briefly, Sf9 supernatants were passed through a Ni_2_-chelated HisTrap Excel column (GE Healthcare) using a variable flow mini-pump (Fisher Scientific). The column was washed with wash buffer (20 mM of sodium phosphate, 0.5 M of NaCl and 20 mM of imidazole [GE Healthcare]) and eluted in 5 ml of elution buffer (20 mM of sodium phosphate, 0.5 M of NaCl, and 500 mM of imidazole).

### Purification of glycoproteins from HEK 293T cells

Anti-His Affinity Resin (GenScript) was used for purification of GP_1,2_s following the manufacturer’s instruction. Briefly, resin was incubated with supernatant and rotated for 30 min and resins were placed into Econo-Pac Disposable Chromatography columns (BioRad, Hercules, CA) and washed with Tris-buffered saline (pH 7.4). Resins were then incubated with hexa-His peptide (GenScript) at a concentration of 0.5 µg/ml. Eluents containing proteins of interest were concentrated using Pierce Protein Concentrator (Thermo Fisher Scientific, Waltham, MA) for downstream use.

### Verification of protein purity

Protein purity was verified via a Colloidal Blue stain, PAS, (ThermoFisher Scientific, Waltham, MA), and western blot. Purified proteins were run on 4–12% Bis-Tris Plus polyacrylamide gels (Life Technologies, Carlsbad, CA). Gels were then stained using PAS to detect GP_1,2_s according to the manufacturer’s instructions. Total protein was then assayed using the Colloidal Blue staining kit (ThermoFisher Scientific, Waltham, MA). Finally, western blots were performed by transferring gels, as above, to iBlot 2 nitrocellulose Mini Stacks (Life Technologies) using the iBlot2 system (Life Technologies).

Membranes were blocked using 5% dry milk in phosphate buffered saline and 0.01% Tween 20 (PBST, Sigma-Aldrich) for 1 h at room temperature. Membranes were then incubated with primary antibody in 5% milk in PBST for 1 h with shaking. Antibody dilutions were as follows: BDBV (rabbit polyclonal, IBT Bioservices, Rockville, MD, USA): 1:5,000; EBOV (6D8, courtesy of John Dye, US Army Medical Research Institute of Infectious Diseases, Frederick, MD): 1:10,000; TAFV (rabbit polyclonal, Alpha Diagnostic International, San Antonio, TX): 1:1,000; SUDV (clone 6D11, BEI Resources, Manassas, VA): 1:5,000; MARV (rabbit polyclonal, IBT Bioservices, Rockville, MD): 1:3,000; and THE^TM^ His Tag Antibody (mouse monoclonal, Genscript). Blots were then washed thrice with PBST and incubated and shook for 1 h at room temperature with secondary antibody. Secondary antibodies were anti-mouse IgG-horseradish peroxidase (HRP) or anti-rabbit IgG-HRP (ThermoFisher Scientific, Waltham, MA), both at 1:5,000 dilution. Blots were then washed three times with PBST, and a 1:1 mixture of Novex ECL HRP Chemiluminescent Substrate (ThermoFisher Scientific, Waltham, MA) was added to the membranes for development using a medical film processor (Konica Minolta Medical Imaging, Wayne, NJ).

### Enzymatic removal of glycans

Glycans were removed from purified GP_1,2_s using PNGase F (New England BioLabs, Beverly, MA) and deglycosylation mix (New England BioLabs, Beverly, MA) according to manufacturer’s instructions. Proteins were imaged using Colloidal Blue analysis, as above, and approximate percentages calculated using Novex Sharp Prestained Protein ladder (ThermoFisher Scientific, Waltham, MA).

### N-glycan release by PNGase F for glycan analysis

Glycan composition analysis was performed by Proteodynamics (Riom, France). N-linked glycans were removed from HEK 293T derived GP_1,2_ using 15 U per 200 µg protein of PNGase F (Promega, Madison, WI, USA) for 15 hours at 37 °C in phosphate buffer (pH of 7.5) while Sf9 cell derived protein was deglycosylated with 15 U of PNGase F (Promega, Madison, WI, USA) and 10 µL of PNGAse A (Sigma) for 15 hours at 37 °C in 50 mM sodium acetate buffer, pH 5.0. To confirm deglycosylation, electrophoresis on NuPAGE 4–12% gels (ThermoFisher Scientific, Waltham, MA) was performed. The N-glycans were then purified and permethylated according to Morelle *et al*.^43^.

### O-glycan release by beta-elimination for glycan analysis

Glycoproteins (200 µg) were neutralized and loaded onto beads and then lyophilized overnight, and then suspended in 200 µL of MilliQ water and 200uL reductive solution [100 mM NaOH, 2 M NaBH4]. Beta-elimination occurred for 15 hours at 45 °C to release O-glycans. Permethylation was performed as for the N-glycans^43^.

### Glycan composition analysis

Once permethylated, purified glycans were solubilized in 20 µL of a 1:1 ratio methanol/DI water mix. A mix of 2 µL non-dilute and 2 µL N-glycans with 2  µL of 2,5-dihydroxybenzoic acid (LaserBio Labs, Sophia-Antipolis, France) matrix solution (10 mg/ml in 1:1 ratio methanol/DI water). Positive ion reflectron MALDI-TOF mass spectra were acquired using an Autoflex III mass spectrometer (Bruker Daltonics, Billerica, MA, USA). The acceleration and reflector voltage conditions were voltage 12 × 1977 V and 90% laser, and the spectra obtained by accumulation of 2,000 shots. The spectra were calibrated with an external standard (PepMix 4, LaserBioLabs, Sophia-Antipolis, France). To elucidate the glycan profiles of the sample, several spectra were obtained from different spots and the values averaged. To interpret the structures of the glycans corresponding to monisotopic masses after deisotoping of the spectra, the EXPAZY GlycoMod tool was used, along with GlycoWorkBench. Relative intensities of glycans were calculated to establish the glycan profile for each spectrum and mean values for the glycan intensities with standard deviations were determined.

### N-glycan site occupancy analysis on EBOV-Makona from Sf9-cells and HEK 293T cells

#### Glycopeptide enrichment method

EBOV/Makona GP_1,2_ proteins from Sf9 and HEK293T (200 µg each) were denatured, reduced by DTT, and alkylated by iodoacetamide and digested by trypsin/Lys-C Mix (Promega, Madison, WI, USA) during 15 hours at 37 °C.according to Rapigest^®^ SF protocol. Glycopeptides were enriched according to ProteoExtract^®^ Glycopeptide Enrichment Kit protocol 72103-3 (Novagen, Madison, WI, USA). 30 µl of glycopeptide sample were added to 150 µl of ZIC Glycocapture Resin (Merck SeQuant AB, Umea, Sweden), and eluted with 225 µl ZIC Elution Buffer (Merck SeQuant AB, Umea, Sweden). The eluted samples were completely evaporated in with a SpeedVac concentrator (ThermoFisher Scientific, Waltham, MA).

#### MALDI-TOF MS analysis of site occupancy

Enriched glycopeptides (15 µl) in 50% methanol were mixed (1:1) with 2,5-dihydroxybenzoic acid (LaserBio Labs, Sophia-Antipolis, FR) matrix solution (10 mg/ml in 1:1 ratio methanol/water). Positive ion linear MALDI mass spectra were acquired on MALDI-TOF/TOF Autoflex Speed (Bruker Daltonics, Billerica, MA, USA). Acquisition conditions used, 10 × 2850 V, laser 25% and 2000 shots.

#### Deglycosylation of the enriched fraction

To remove glycans from the peptides, 5 µl of suspended glycopeptides were adjusted to 10 mM sodium acetate buffer pH 5 and 1 µl of PNGase A and 0.7 µl of PNGase F (Promega, Madison, WI, USA) or PNGase A (for insect cell protein) were added to deglycosylate during 15 hours at 37 °C. The deglycosylated peptides after preparation on Zip Tips C18 (EMD Millipore, Billerica, MA, USA) were mixed (1:1) with CHCA (LaserBio Labs, Sophia-Antipolis, France) matrix solution (7 mg/ml 50:50 acetonitrile/water, 0.1% TFA). Positive ion reflectron MALDI mass spectra were acquired on MALDI-TOF/TOF Autoflex Speed (Bruker Daltonics, Billerica, MA, USA). Acquisition conditions were 10 × 1950 V, laser 30% with 2000 shots.

## Electronic supplementary material

Supplementary Information
