# Supplementary material for: Production and Purification of Filovirus Glycoproteins in Insect and Mammalian Cell Lines
Source: Sci Rep. 2017 Nov 8;7:15091. doi: 10.1038/s41598-017-15416-3 (PMC5678155; doi:10.1038/s41598-017-15416-3)
Supplement: Supplementary file 1 — Supplementary Information [file 41598_2017_15416_MOESM1_ESM.pdf]

Production and Purification of Filovirus Glycoproteins in Insect and Mammalian Cell Lines

Elizabeth C. Clarke[a], Amanda L. Collar[a], Chunyan Ye[a], Yíngyún Caì[b], Eduardo Anaya[c], Derek Rinaldi[c], Britney Martinez[d], Sarah Yarborough[d], Christine Merle[e], Manfred Theisen[e], Jiro Wada[b], Jens H. Kuhn[b], and Steven B. Bradfute[a,*]

a. Center for Global Health, Division of Infectious Diseases, Department of Internal Medicine, University of New Mexico, Albuquerque, New Mexico 87131, USA
b. Integrated Research Facility at Fort Detrick, National Institute of Allergy and Infectious Diseases, National Institutes of Health, Frederick, Maryland 21702, USA
c. Department of Pathology, University of New Mexico, Albuquerque, New Mexico 87131, USA
d. Undergraduate Pipeline Network, University of New Mexico, Albuquerque, New Mexico 87131, USA.
e. Proteodynamics SARL, Riom, France.

Supplementary Information

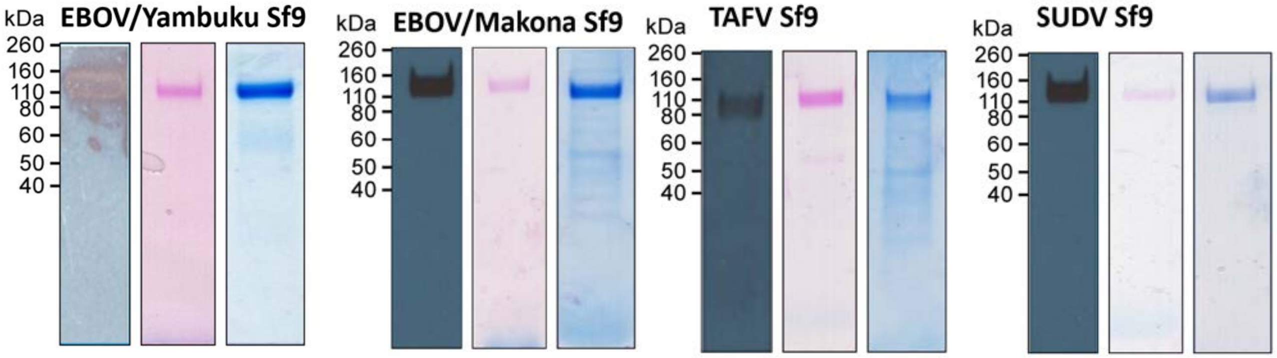

Supplemental Figure S1. Western blot, colloidal blue and PAS images for EBOV/Yambuku, EBOV/Makona, SUDV and TAFV glycoproteins from Sf9 cells.

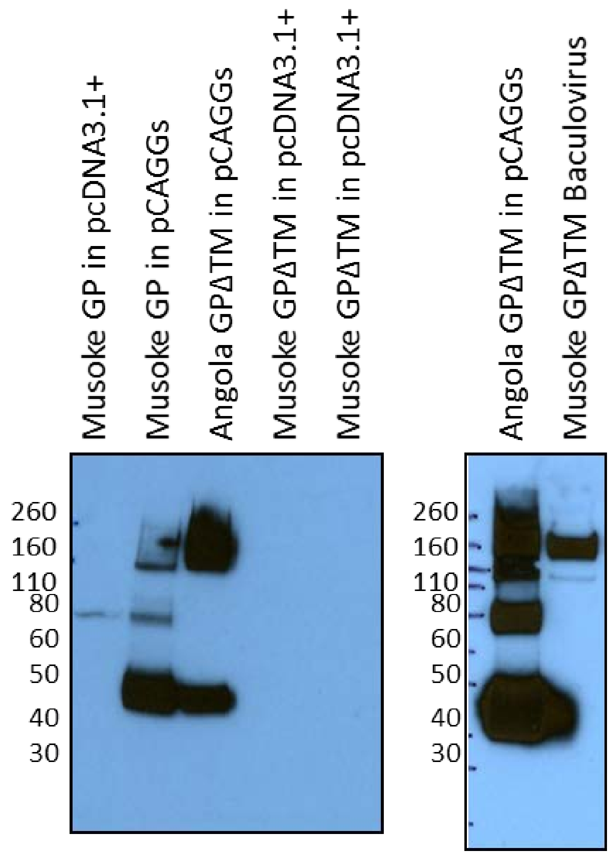

Supplemental Figure S2. Western blots of MARV $GP_{1,2}$ expression. From left, Panel A: MARV/Musoke GP (full length) in pcDNA3.1+ expressed in HEK 293T cells; MARV/Musoke GP (full length) in pCAGGs expressed in HEK 293T cells; MARV/Angola GPΔTM in pCAGGs expressed in HEK 293T cells; MARV/Musoke GPΔTM (1–648) in pcDNA3.1+ expressed in HEK 293T cells; MARV/Musoke GPΔTM (1-636) in pcDNA3.1+ expressed in HEK 293T cells. Panel B: MARV/Angola GPΔTM in pCAGGs expressed in HEK 293T cells (repeat); MARV/Musoke GPΔTM (1-648) in baculovirus expressed in Sf9 cells.

Supplemental Figure S3. Full size gels and blots for cropped images/lanes shown. For each filovirus glycoprotein, a PAS, colloidal blue stain and a western blot are shown. All protein ladders are Novex Sharp pre-stained protein standards (Thermofisher). Lanes shown in figures are labelled in bold.

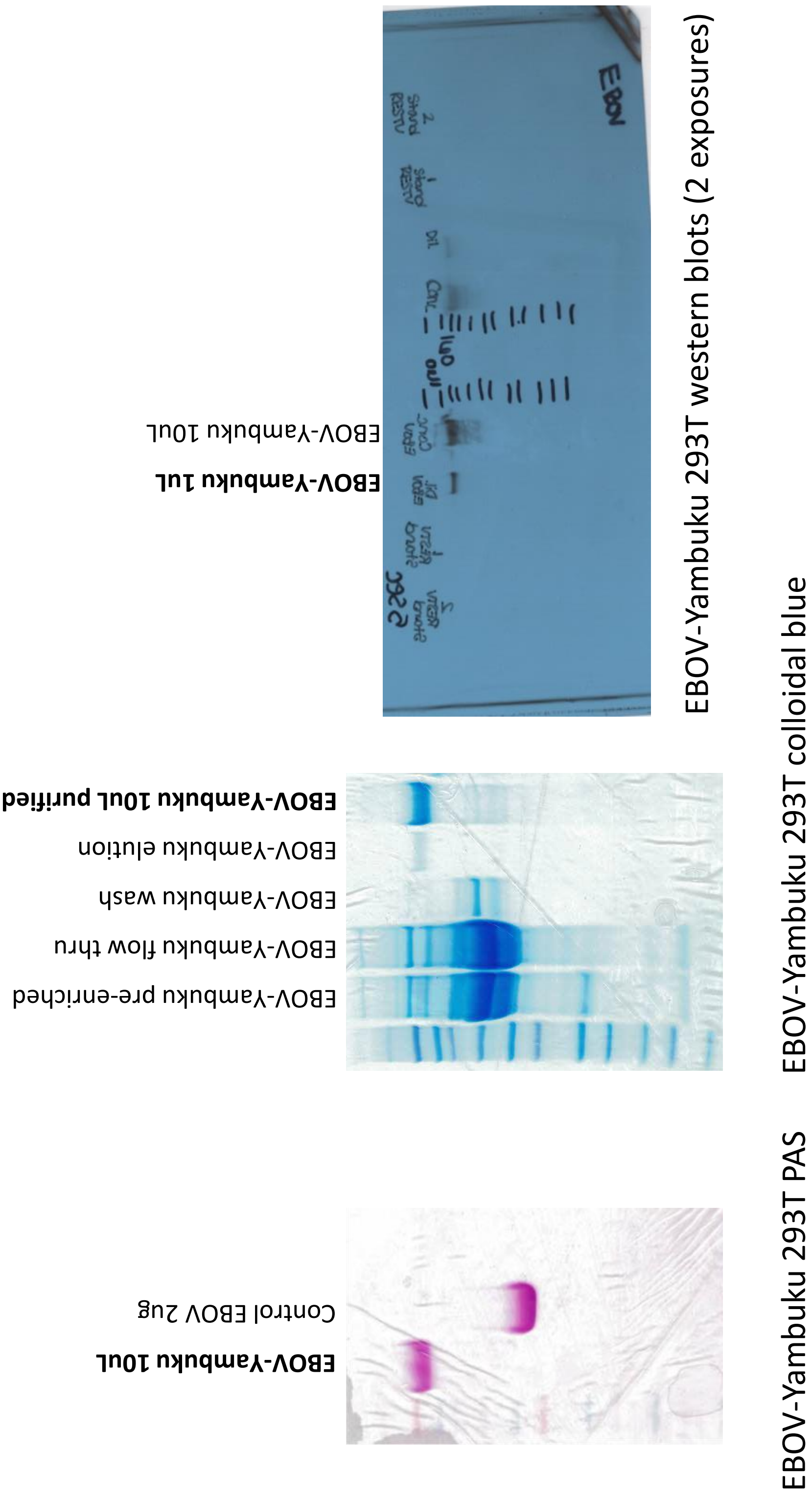

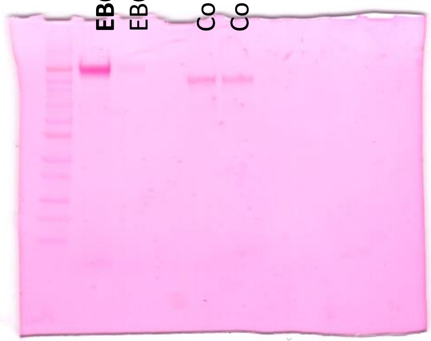

EBOV-Makona 293T PAS

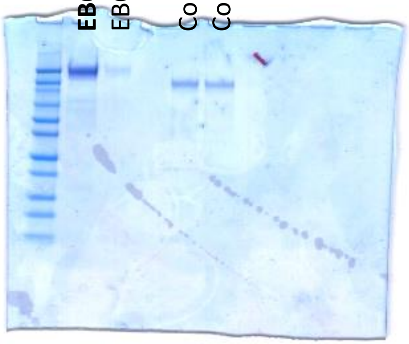

EBOV-Makona 293T colloidal blue

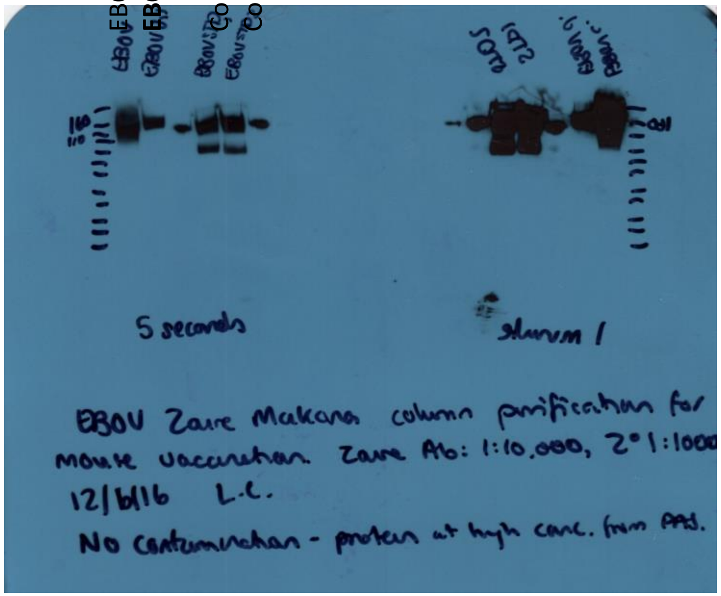

EBOV-Makona 293T western blot (2 exposures)

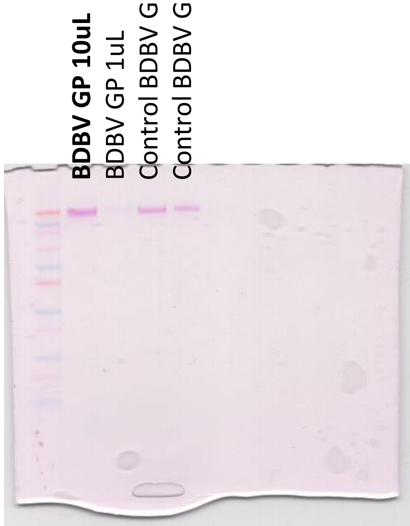

BDBV 293T PAS

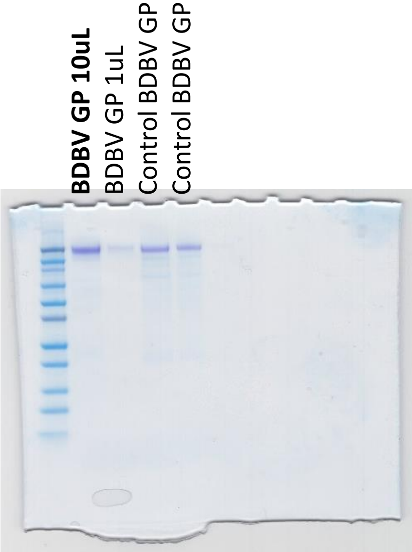

BDBV 293T colloidal blue

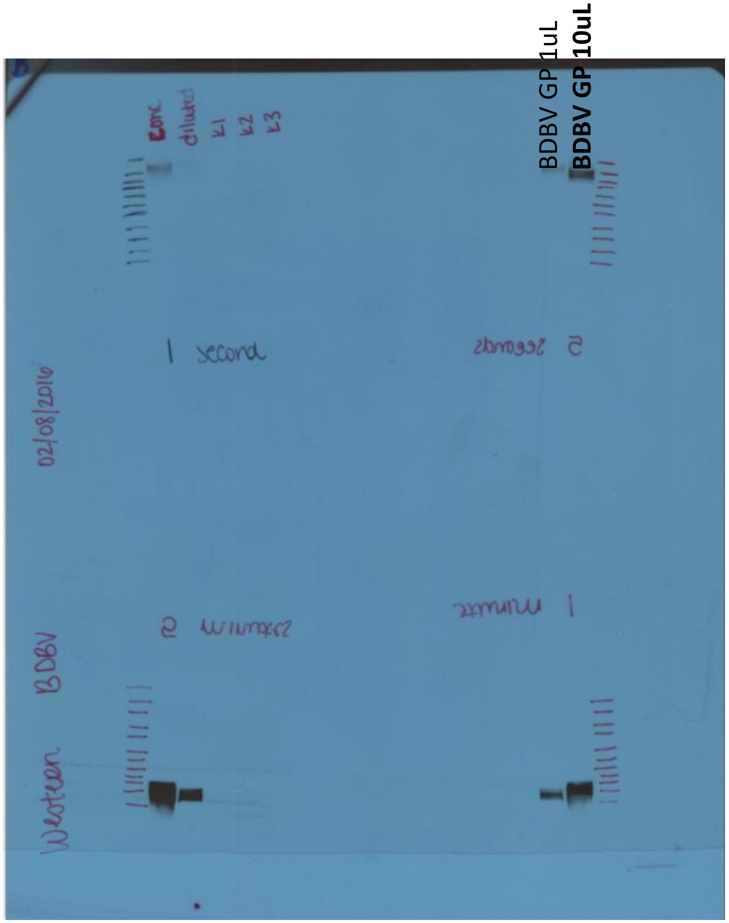

BDBV 293T western blot (4 exposures)

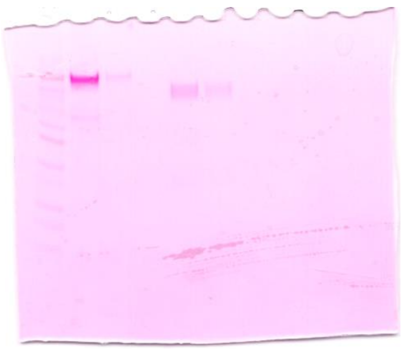

SUDV GP 293T PAS

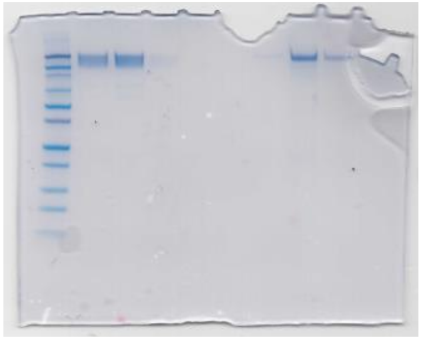

SUDV GP 293T colloidal blue

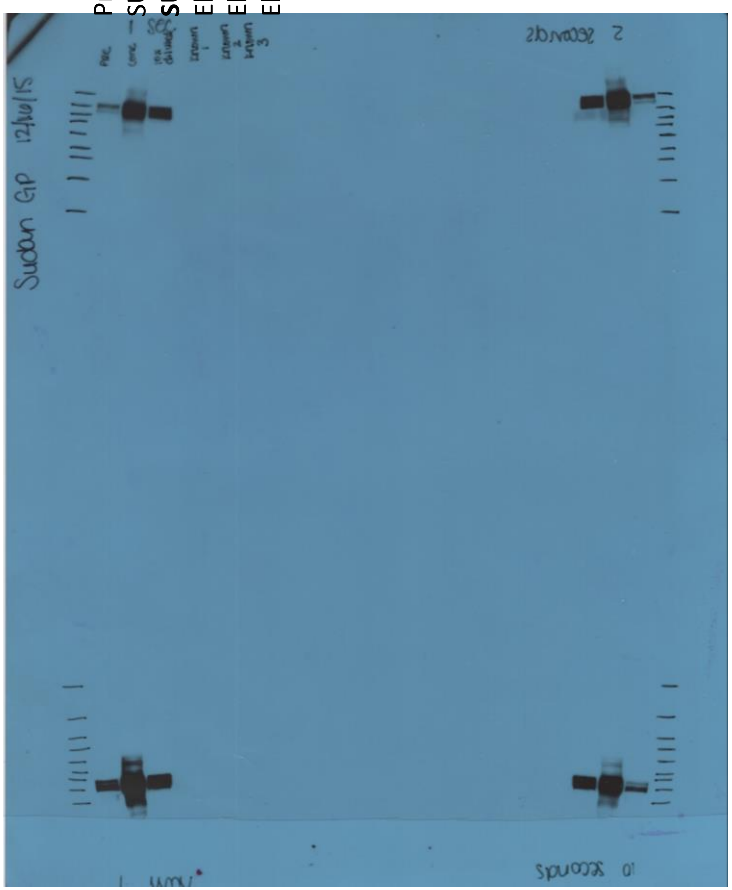

SUDV GP 293T western blot (4 exposures)

TAFV GP 10uL
TAFV GP 1uL
Control TAFV GP
Control TAFV GP

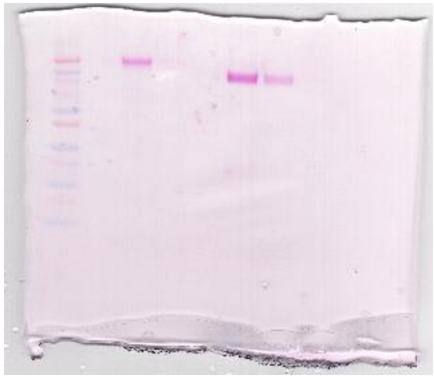

TAFV GP 293T PAS

TAFV GP 10uL
TAFV GP 1uL
Control TAFV GP
Control TAFV GP

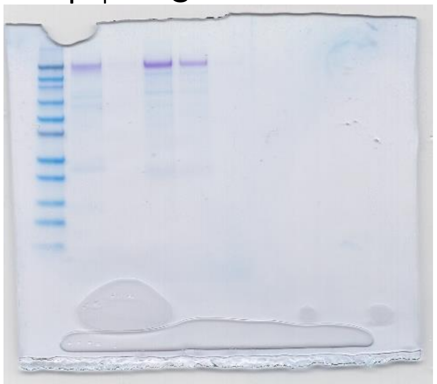

TAFV GP 293T colloidal blue

TAFV GP 10uL
TAFV GP 1uL

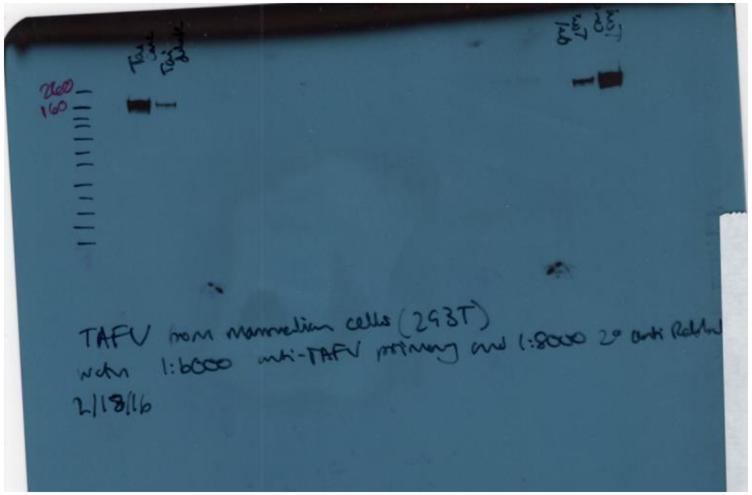

TAFV GP 293T western blot (2 exposures)

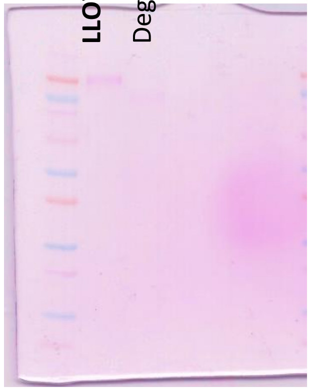

LLOV GP 293T PAS

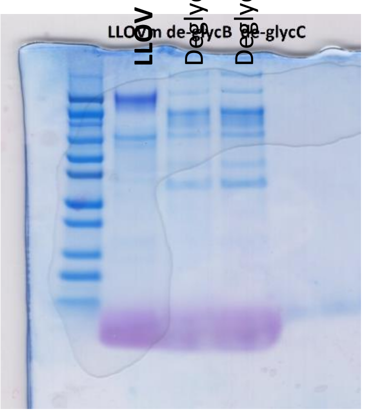

LLOV GP 293T colloidal blue

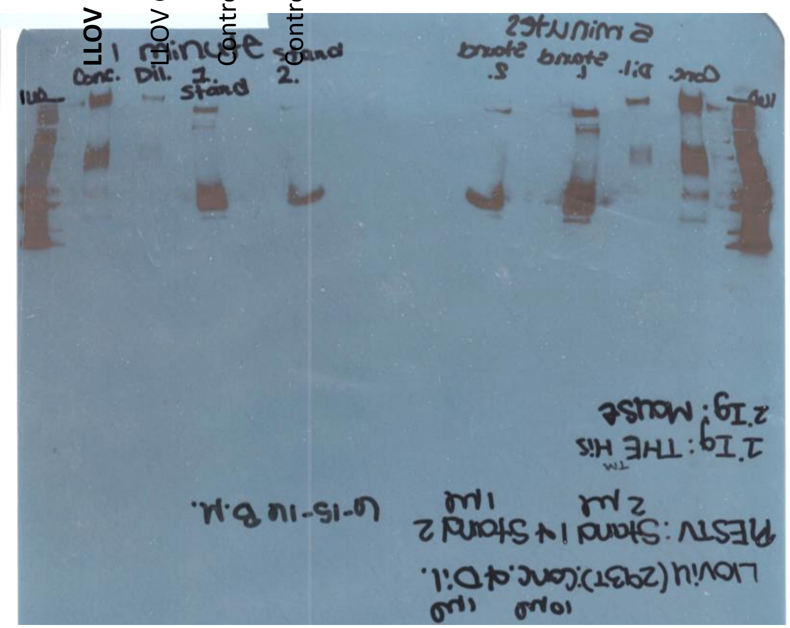

LLOV GP 293T western blot (2 exposures)

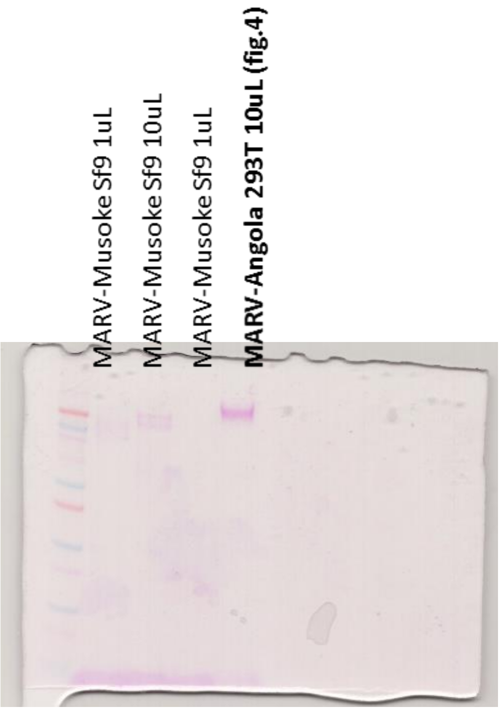

MARV-Angola GP 293T PAS

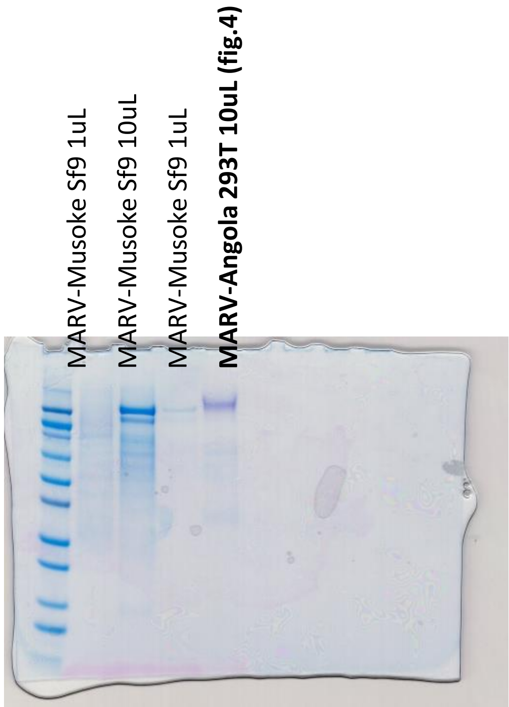

MARV-Angola GP 293T colloidal blue

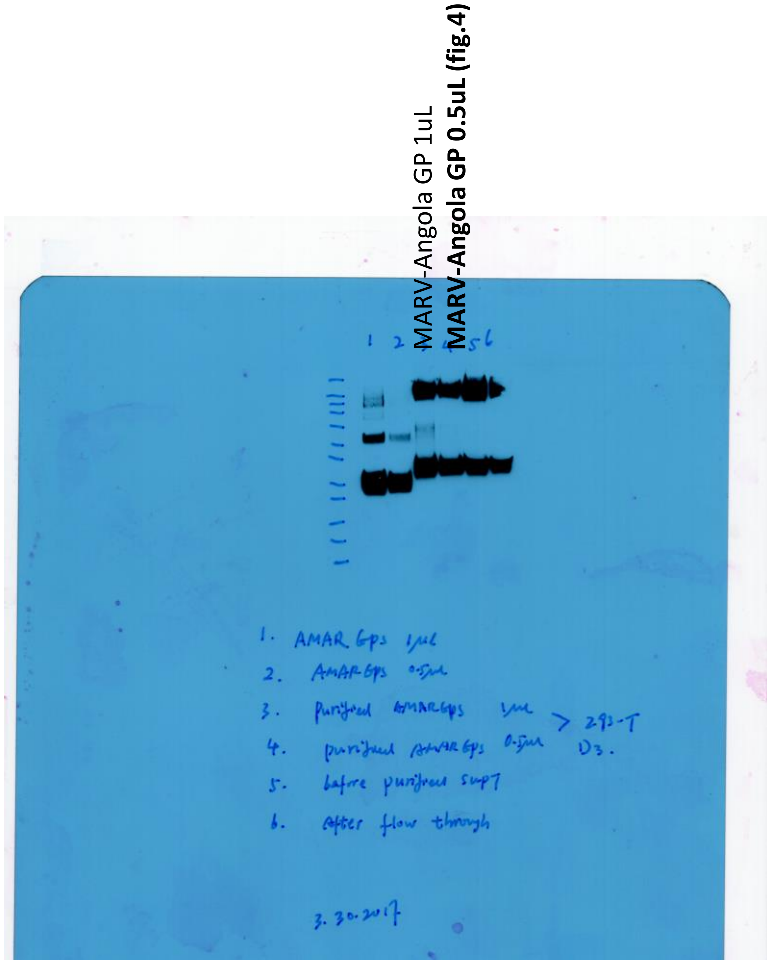

MARV-Angola GP 293T western blot

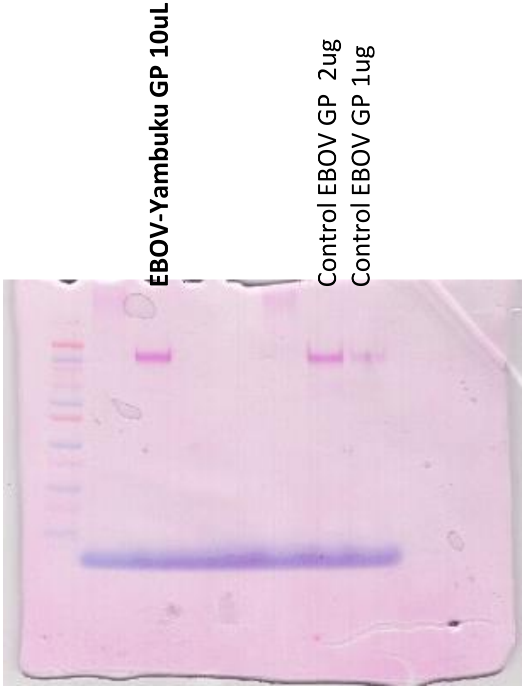

EBOV-Yambuku Sf9 PAS

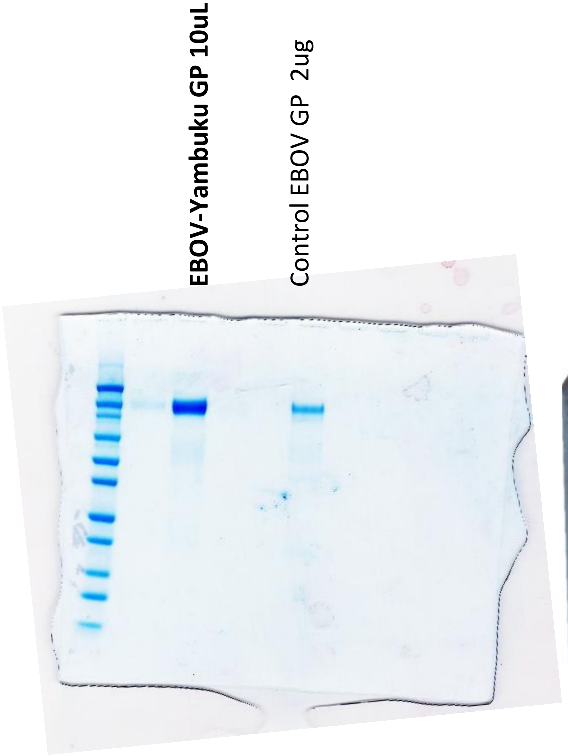

EBOV-Yambuku Sf9 colloidal blue

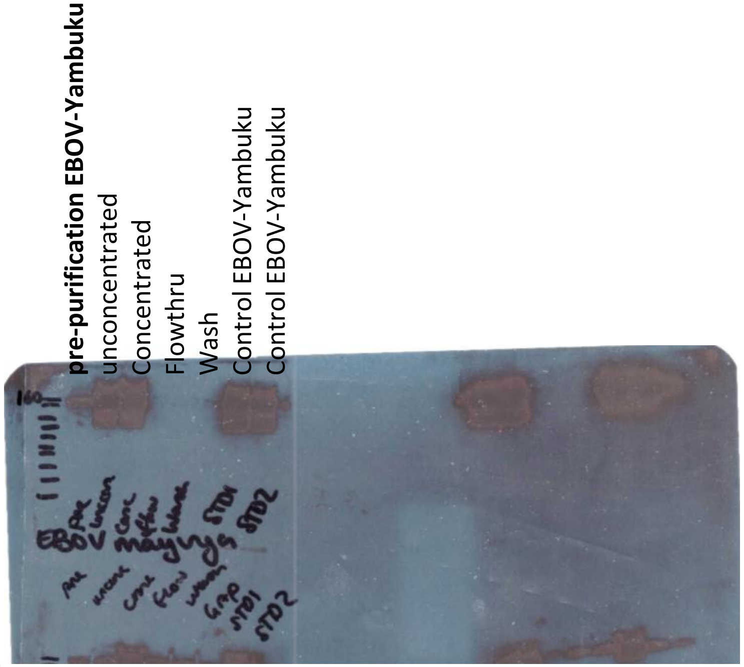

EBOV-Yambuku Sf9 western blot

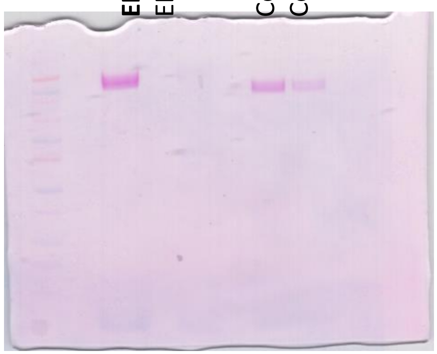

EBOV-Makona GP Sf9 PAS

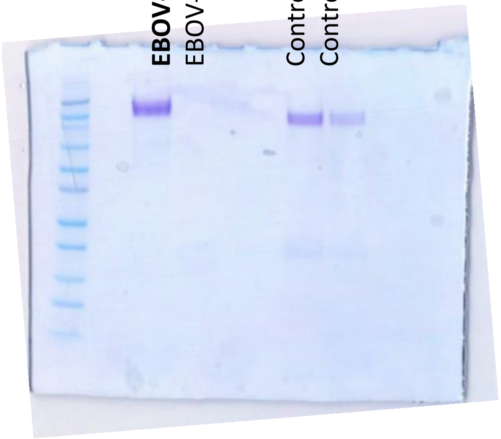

EBOV-Makona GP Sf9 colloidal blue

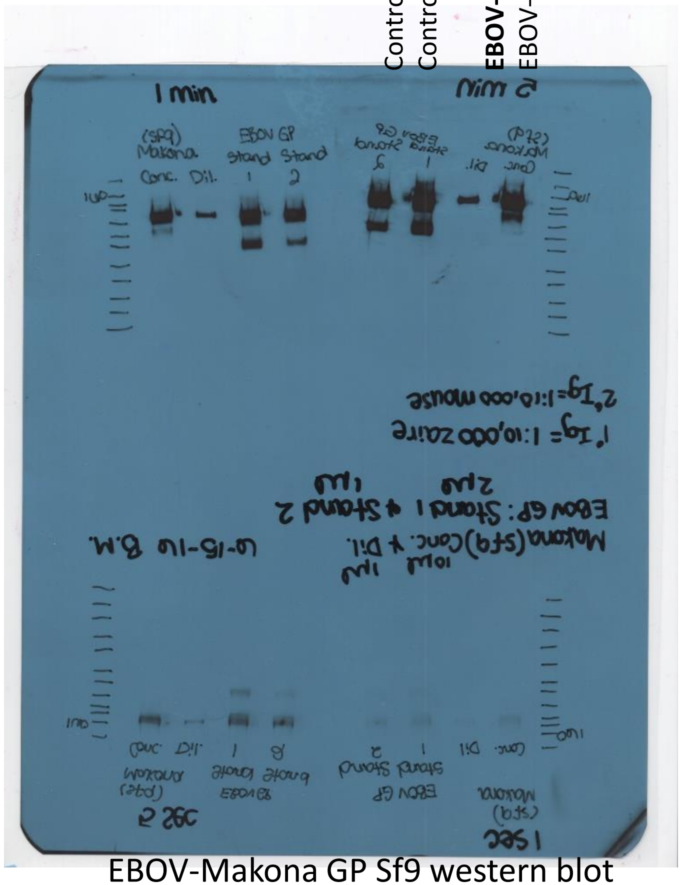

EBOV-Makona GP Sf9 western blot

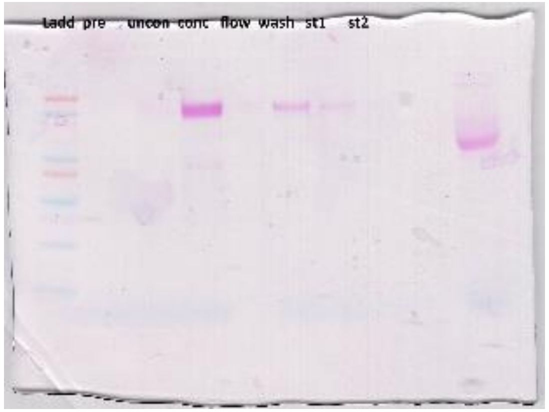

TAFV GP Sf9 PAS

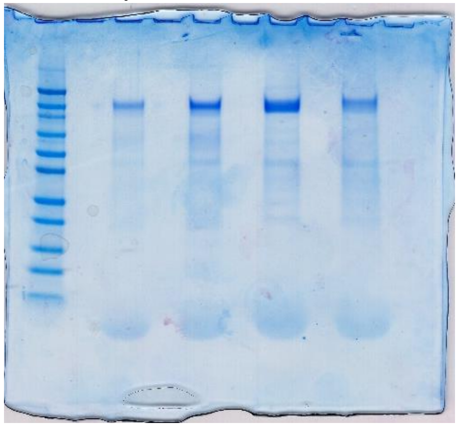

TAFV GP Sf9 colloidal blue

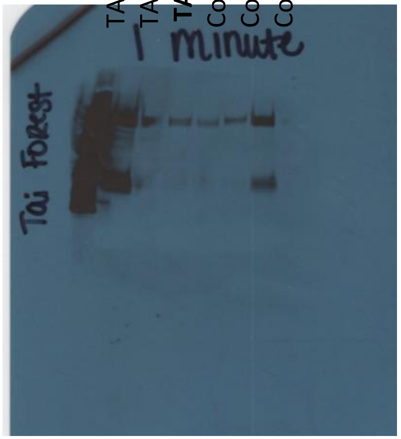

TAFV GP Sf9 western blot

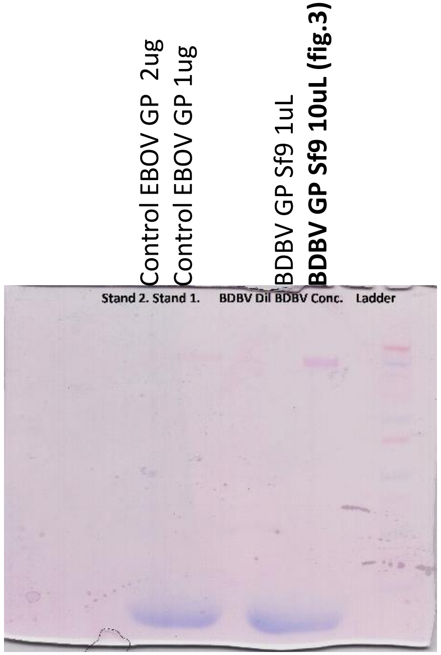

BDBV GP Sf9 PAS

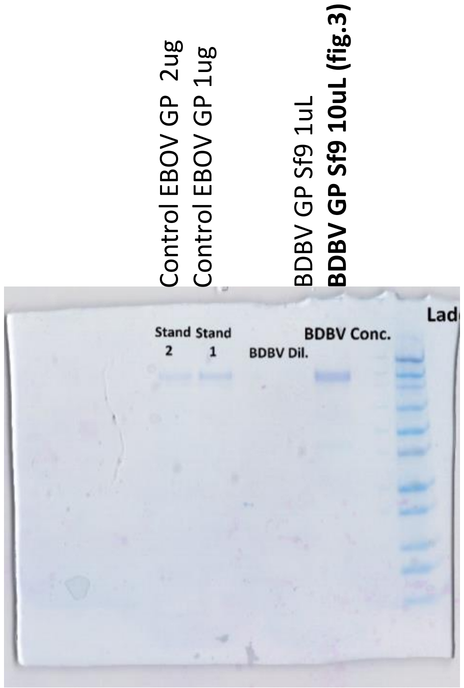

BDBV GP Sf9 colloidal blue

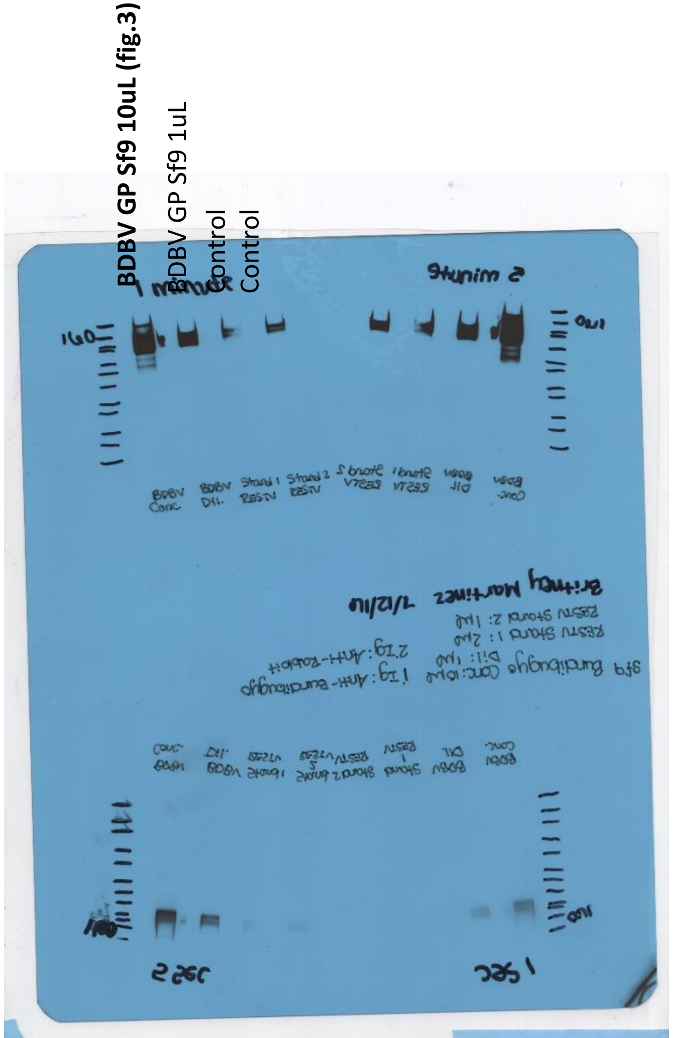

BDBV GP Sf9 western blot

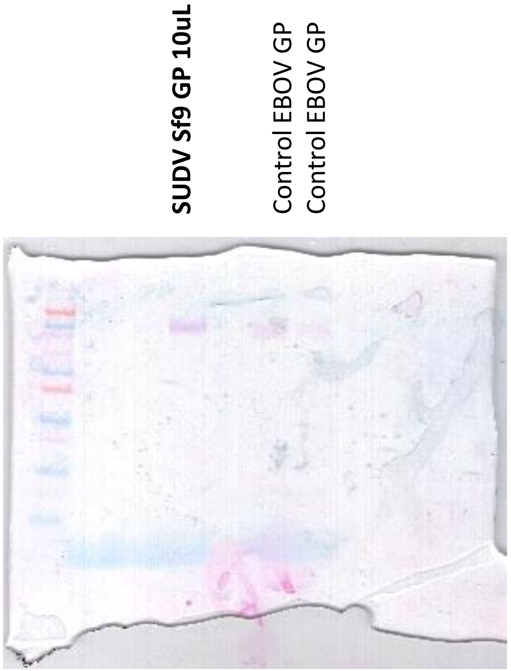

SUDV GP Sf9 PAS

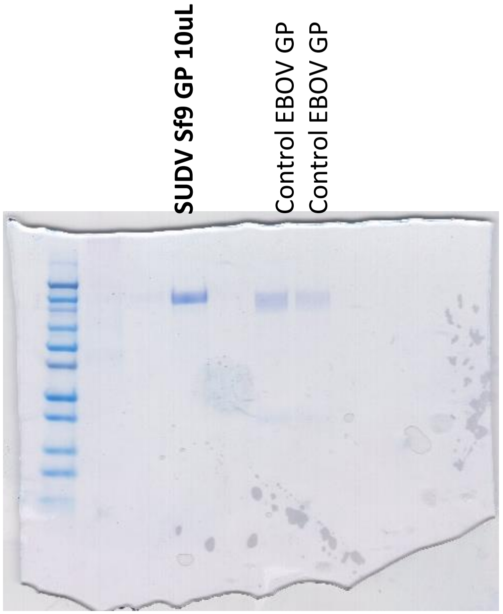

SUDV GP Sf9 colloidal blue

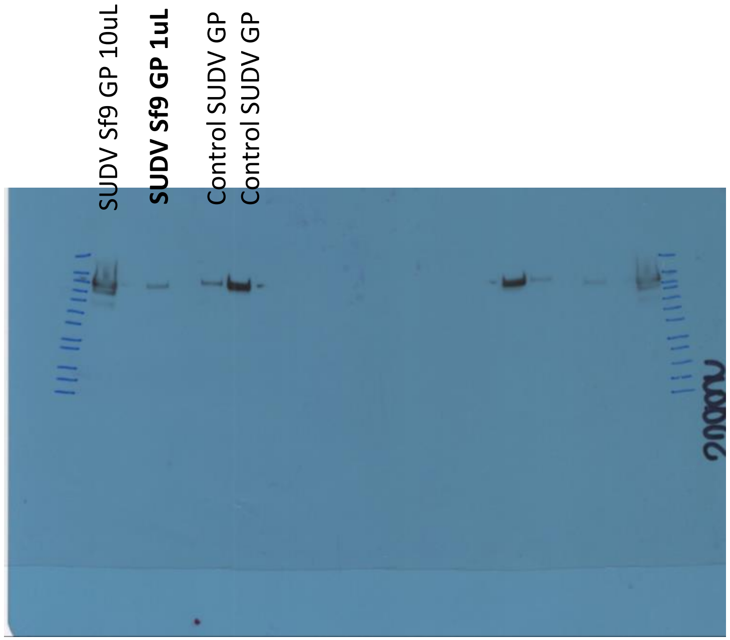

SUDV GP Sf9 western blot

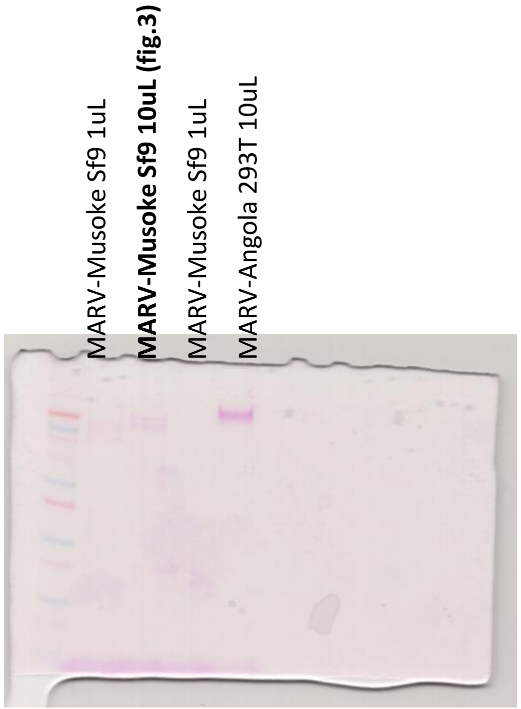

MARV-Musoke GP Sf9 PAS

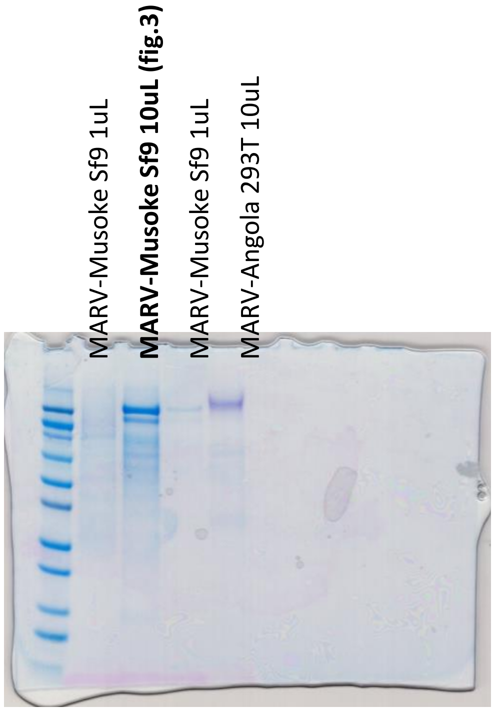

MARV-Musoke GP Sf9 colloidal blue

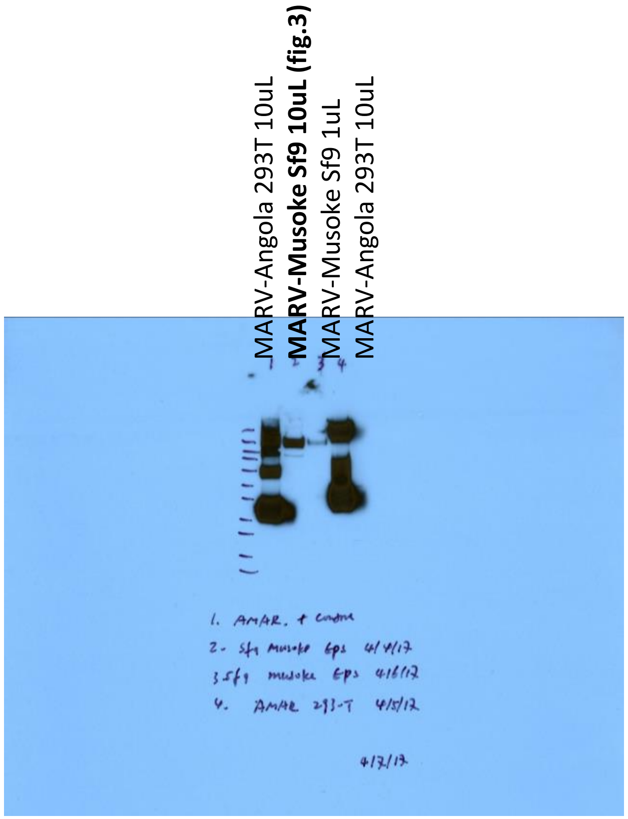

MARV-Musoke GP Sf9 western blot
